# Discovery of Transcription Factors and Regulatory Regions Driving *In Vivo* Tumor Development by ATAC-seq and FAIRE-seq Open Chromatin Profiling

**DOI:** 10.1371/journal.pgen.1004994

**Published:** 2015-02-13

**Authors:** Kristofer Davie, Jelle Jacobs, Mardelle Atkins, Delphine Potier, Valerie Christiaens, Georg Halder, Stein Aerts

**Affiliations:** 1 Laboratory of Computational Biology, Center for Human Genetics, KU Leuven, Leuven, Belgium; 2 Laboratory of Growth Control and Cancer Research, Center for Human Genetics, KU Leuven, Leuven, Belgium; 3 VIB Center for the Biology of Disease, Laboratory for Molecular Cancer Biology, Leuven, Belgium; St Jude Children's Research Hospital, United States of America

## Abstract

Genomic enhancers regulate spatio-temporal gene expression by recruiting specific combinations of transcription factors (TFs). When TFs are bound to active regulatory regions, they displace canonical nucleosomes, making these regions biochemically detectable as nucleosome-depleted regions or accessible/open chromatin. Here we ask whether open chromatin profiling can be used to identify the entire repertoire of active promoters and enhancers underlying tissue-specific gene expression during normal development and oncogenesis in vivo. To this end, we first compare two different approaches to detect open chromatin in vivo using the Drosophila eye primordium as a model system: FAIRE-seq, based on physical separation of open versus closed chromatin; and ATAC-seq, based on preferential integration of a transposon into open chromatin. We find that both methods reproducibly capture the tissue-specific chromatin activity of regulatory regions, including promoters, enhancers, and insulators. Using both techniques, we screened for regulatory regions that become ectopically active during Ras-dependent oncogenesis, and identified 3778 regions that become (over-)activated during tumor development. Next, we applied motif discovery to search for candidate transcription factors that could bind these regions and identified AP-1 and Stat92E as key regulators. We validated the importance of Stat92E in the development of the tumors by introducing a loss of function Stat92E mutant, which was sufficient to rescue the tumor phenotype. Additionally we tested if the predicted Stat92E responsive regulatory regions are genuine, using ectopic induction of JAK/STAT signaling in developing eye discs, and observed that similar chromatin changes indeed occurred. Finally, we determine that these are functionally significant regulatory changes, as nearby target genes are up- or down-regulated. In conclusion, we show that FAIRE-seq and ATAC-seq based open chromatin profiling, combined with motif discovery, is a straightforward approach to identify functional genomic regulatory regions, master regulators, and gene regulatory networks controlling complex in vivo processes.

## Author Summary

The functional expression of all genes is regulated by proteins, namely transcription factors that bind to specific areas of DNA known as regulatory regions. Whereas most DNA in our genome is normally bound by other proteins (histones) and packaged into units called nucleosomes, a specific subset of tissue-specific regulatory regions is responsible for tissue-specific gene expression; these active regions are nucleosome-depleted and bound by transcription factors. We use two techniques to identify these open chromatin regions, in a normal tissue and a Ras^V12^ induced cancer tissue. We discovered a remarkable change in the accessible regulatory landscape between these two tissues, with several thousand regions becoming more accessible in the cancer tissue. We identified two transcription factors known to be involved in cancer (AP-1 and Stat92E) controlling these newly accessible regulatory regions. Finally, we introduced a mutation resulting in Stat92E becoming non-functional in the cancer tissue, which decreased the severity of the tumor. Our study shows that open chromatin profiling can be used to identify complex in vivo processes, and we shed new light on Ras dependent cancer development.

## Introduction

Gene expression in higher eukaryotes is tightly controlled by complex *cis*-regulatory systems consisting of multiple enhancers modulating the transcription levels of a target gene. Mapping all active promoters and enhancers in a cell type provides an entry point to reverse engineer the functional gene regulatory network and to decipher the genomic *cis*-regulatory logic underlying a cell type’s functional transcriptome. Understanding changes in regulatory landscapes between different cell types, for example during cellular differentiation, or between normal and diseased cellular states, can furthermore provide a bridge between the genomic sequence and emerging transcriptome changes. Recent advances in chromatin studies have uncovered several characteristic features of active and repressed chromatin within and around regulatory regions. A typical active promoter or enhancer in higher eukaryotes is depleted for nucleosomes over relatively large regions of up to hundreds of base pairs, often spanning the entire enhancer length [1]. In addition, nucleosomes flanking active regulatory regions usually carry histone modifications, such as H3K27Ac and H3K4me1 [2], and in human and other vertebrates, active promoters and enhancers may have dynamic hyper- or hypomethylated CpG dinucleotides, denoting inactive and active enhancers respectively, whereby changes in methylation usually accompany changes in activity [3]. All of these features, to some extent, correlate significantly with the expression of the target gene(s) that they control [1,4]. Furthermore, these features can be exploited to identify regulatory regions at a genome-wide scale, for example, integrative methods have been developed based on Hidden Markov Models that accurately predict various types of regulatory regions using particular combinations of histone modifications [5]. Another, more recently discovered feature of active enhancers is that RNA, known as eRNA, is transcribed from them in a bidirectional way by RNApolII [4]. This property was used in the Fantom project and lead to the identification of ∼44,000 tissue and cell type specific enhancers in the human genome. However, the technique that has been primarily used to map regulatory landscapes across hundreds of human cell lines, notably in the ENCODE and Epigenomics Roadmap projects, is the detection of open chromatin using DNaseI hypersensitivity coupled with high throughput sequencing (DNaseI-seq) [1,6,7]. DNaseI-seq identifies regions by fragmenting chromatin with the DNase I enzyme, an endonuclease that randomly cleaves regions of accessible DNA, and for which cleavage is hindered by the presence of nucleosomes. This cleavage results in an increased number of cut sites in nucleosome-depleted regions, generating fragments with smaller sizes, allowing their enrichment based on size-selection, before high-throughput sequencing. As such, DNaseI-seq has mapped genome-wide regulatory element across many human and mouse cell types, as well as across developmental stages during Drosophila embryogenesis [8]. DNaseI-seq however, has some limitations as it requires large amounts of input material and has a complicated procedure. Consequently, it has been mainly applied to *in vitro* cell cultures and cancer cell lines and has seen limited applications to in vivo post-embryonic biological systems. For example, it is generally not possible with this approach to assay to what extent the open chromatin profile of cells within a tumor, or during tumor development, is altered. It is indeed conceivable that the joint interpretation of cancer genomes and cancer transcriptomes, both of which can be sequenced directly from tumor biopsies down to the single cell level, will require the intermediate layer of the “functional genome” to permit us to understand how the epigenomic changes drive changes in gene expression. Therefore, to profile this chromatin layer, alternative approaches are required to progress towards smaller cell populations.

Two alternative methods for open chromatin profiling were recently developed which overcome the limitation of sample size, namely FAIRE-seq (Formaldehyde Assisted Isolation of Regulatory Elements) [9], and ATAC-seq (Assay for Transposase Accessible Chromatin) [10]. We wanted to know whether these methods are both suitable to identify the functional regulatory elements operating in a wild type tissue or in a tumor. FAIRE-seq relies on the separation of open versus closed chromatin by phenol-chloroform extraction, whereby fragments with high nucleosome content are captured in the organic phase, and sequencing libraries are prepared from the aqueous phase [9]. FAIRE-seq derived genome-wide enhancer maps, although noisier than DNaseI-seq have been shown to be highly correlated with DNaseI-seq [11]. FAIRE-seq has recently been applied successfully to identify differential enhancer usage between multiple Drosophila tissues and developmental time points, finding thousands of enhancers that change activity during development [12]. The most recent method, ATAC-seq, uses a bacterial (Tn5) transposase, an enzyme that inserts a short fragment of DNA (a transposon) into another molecule of DNA, or in this case, inserts two short fragments separate from each other [10]. As the transposase is unable to access DNA that is either bound by nucleosomes or strongly bound transcription factors, it integrates its transposons preferentially into accessible or open chromatin. In the case of ATAC-seq the transposase inserts two fragments of DNA which act as tags, and a mechanism to fragment the DNA (as they are inserted ∼9 base pairs apart), a process known as tagmentation [13]. Buenrostro et al. recently showed that ATAC-seq was able to accurately identify the nucleosome free regions of a lymphoblastoid cell line (GM12878), and the authors were able to obtain profiles comparable to DNaseI-seq in signal to noise ratio and specificity from much lower quantities of cells (100–10000 fold less) than generally used for DNaseI-seq [10]. This technique is thus promising to apply to dissected tissues, for example by micro-dissection of tumor samples, sorting of low populations of cells or other low-input samples.

In this study we compare FAIRE-seq and ATAC-seq to discover active regulatory regions in a wild type tissue and during tumor development, and compare both approaches in terms of signal-to-noise ratio, accuracy of enhancer identification, resolution to recover TF footprints, and ability to identify changes during cellular state transitions, such as during oncogenesis. As a model system, we have used the developing Drosophila eye on the one hand, and a genetically induced tumor model in the developing Drosophila eye on the other hand. The tumor model is based on the over-expression of oncogenic Ras^V12^ combined with the loss of the cell polarity gene *scribble* (*scrib*), and is a well-established model to study Ras-dependent oncogenesis in vivo [14,15]. The combination of Ras^V12^ and *scrib* ^*-/-*^ mutations cause differentiation defects, coupled with over-proliferation and invasion of the surrounding tissue [16]. Whether such a severe phenotypic change is driven by transcription factors operating within a stable, unchanged chromatin state, or whether changing gene expression profiles are accompanied by widespread changes in the regulatory landscape, has not yet been investigated. Given the similarity between this invasive cancer model and the mesenchymal state transitions occurring in human epithelial cancers, such as epithelial-to-mesenchymal transition, further insight into chromatin modulation in this model system may also be relevant to understand regulatory changes during human oncogenic processes.

Our results suggest that open chromatin profiling can provide valuable and previously unobtainable information crucial for understanding how regulatory information is encoded in the genome and how regulatory programs determine phenotype and behavior in vivo.

## Results

### ATAC-seq and FAIRE-seq identify regulatory regions with active chromatin in Drosophila eye development

**Fig 1:**
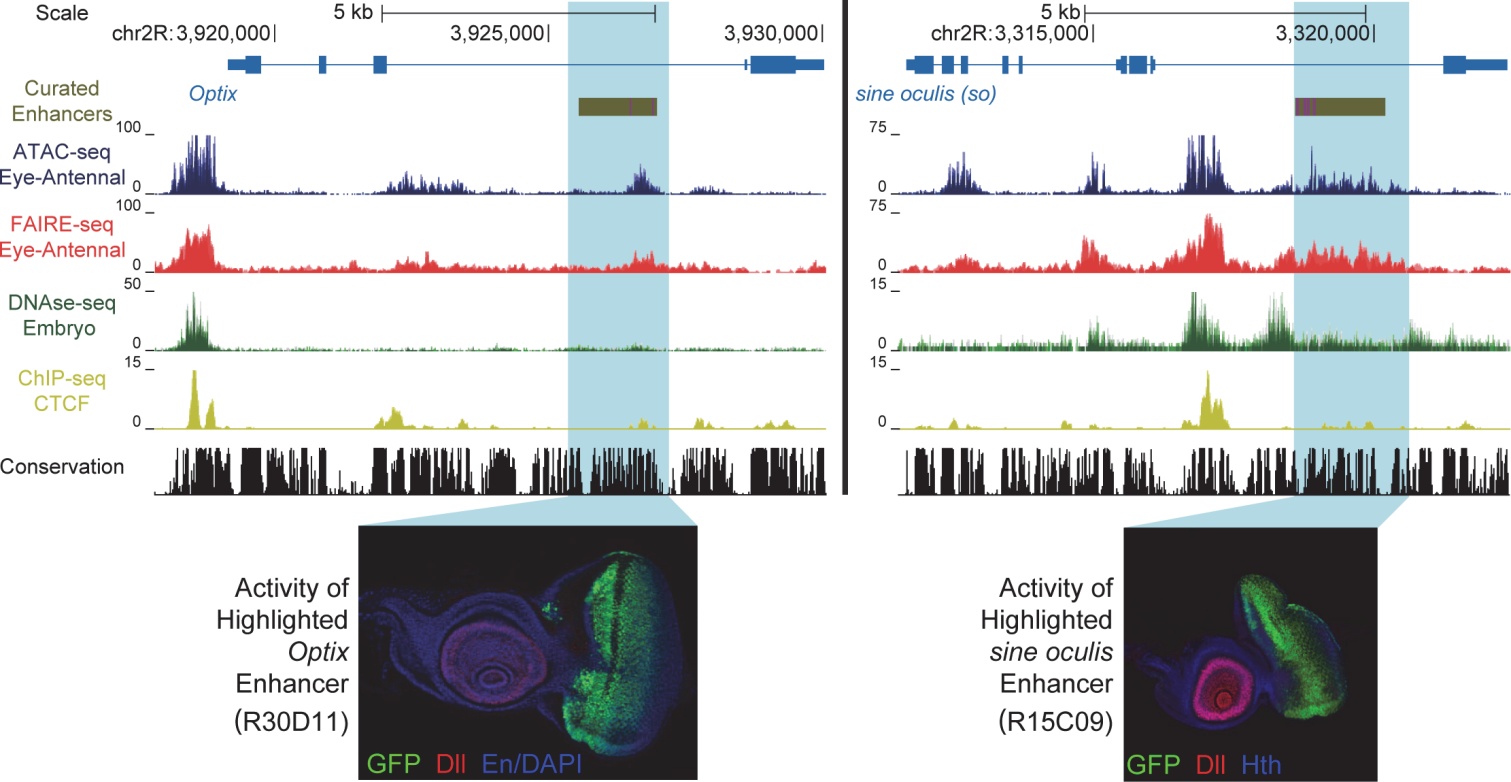
Enhancer identification in the eye primordium by ATAC-seq and FAIRE-seq. Two example eye-related genes from the *Drosophila melanogaster* genome (dm3). Tracks shown include: experimentally validated enhancers from ORegAnno and REDFly [72,73] (shown in Gold), coverage data in the eye-antennal disc for ATAC-seq (Blue), FAIRE-seq (Red) and CTCF ChIP (Yellow), DNaseI-seq (Green) from whole stage 11 embryos [8] and finally sequence conservation across 14 Drosophila species (Black). On the left the gene locus of *Optix* is shown. The region in gold is a known enhancer with binding sites for Eyeless overlaid in purple, with FlyLight enhancer activity of R30D11 below. This region is identified as a peak in both ATAC-seq and FAIRE-seq in the eye, but not in embryonic DNaseI-seq. On the right is the gene locus of *sine oculis* (*so*). The region in gold is a known enhancer with binding sites for Eyeless and Twin of Eyeless overlaid in purple. The reporter activity of this enhancer (R15C09) is shown by the Janelia Farm FlyLight project. Again, this enhancer is identified as a peak in both ATAC-seq and FAIRE-seq but not in embryonic DNaseI-seq. In both examples, peaks can be seen in the CTCF ChIP-seq data corresponding to regions identified as open by all three other techniques. Some small differences between ATAC-seq and FAIRE-seq can also be seen, including a higher background signal for FAIRE-seq. Images obtained from the FlyLight database (http://flweb.janelia.org/cgi-bin/flew.cgi).

**Fig 2:**
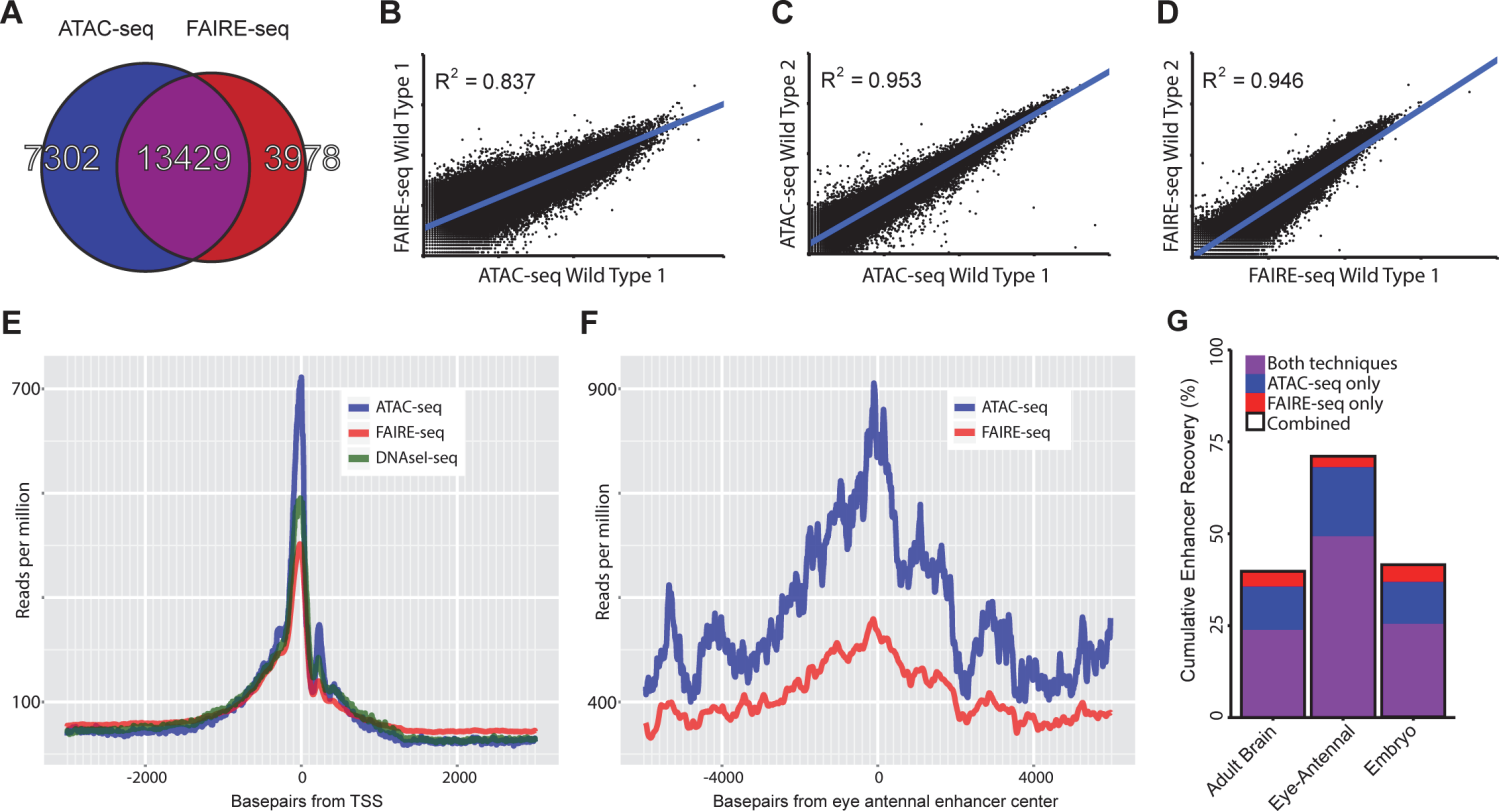
Comparing general features of ATAC-seq and FAIRE-seq. (A) Venn diagram of peaks called for each technique and the overlap between techniques. (B-D) Correlation plots for replicates of both techniques showing normalized read counts for the entire genome split into regions based on conservation [32]. Both techniques correlate very well within techniques (r^2^ ∼ 0.95) despite each wild type sample being from different *D*.*melanogaster* genetic backgrounds. Correlation between the techniques on the same sample was 0.837; in this case FAIRE-seq seems to reach a limit of signal strength before ATAC-seq does. (E) Aggregation plot showing the aggregated signal for each technique across all transcription start sites (TSS) throughout the genome with a 2.5kb window on either side. Although each technique is enriched exactly at the TSS, there are large differences in the size of this peak, despite being normalized to the total read count within these regions. Note that the background level of FAIRE-seq is higher than that of ATAC-seq and DNaseI-seq which are roughly equal, indicating increased noise level in FAIRE-seq. (F) Aggregation plot showing the aggregated signal for ATAC-seq and FAIRE-seq across all Janelia Farm eye-antennal enhancers with a 5kb window on either side. A more distinct peak can be seen in ATAC-seq which also has a higher level overall, due to the fact that it recovers more enhancers than FAIRE-seq. (G) Sets of enhancers known to be expressed in specific tissues were extracted from the FlyLight database [21], these were then compared with the peaks identified from our ATAC-seq and FAIRE-seq data. The set with the most number of enhancers recovered was the eye-antennal enhancers with a total of 71.01% recovered, whilst 49.31% were recovered by both techniques, an extra 18.75% were identified by ATAC-seq only and 2.95% were identified by FAIRE-seq only.

The larval eye-antennal disc of *Drosophila melanogaster* is a widely used model system for the study of spatio-temporal gene regulation and cellular differentiation. This is because in one tissue both dimensions of "time and space" are present, with cells in many different states, from pluripotent cells and specified neuronal precursors, to the lineage committed sensory neurons and accessory cells [17–20]. To map the plethora of regulatory regions operating in all of these cell types, and to simultaneously assess the performance of different biochemical approaches to identify accessible regions, we applied both ATAC-seq and FAIRE-seq to several different genotypes of wild type eye-antennal discs (see Materials and Methods) (Fig. 1). When looking at well-known master regulators of eye development, such as *Optix* and *sine oculis* (*so*), we found both methods to yield highly reproducible results whereby the open chromatin peaks mark previously known enhancers within these genes (Fig. 1). Peaks called by both methods highly overlap (Fig. 2A), and the normalized peak heights between the methods are strongly correlated (Fig. 2B-D). Both methods show a similar distribution of peaks within promoters, intronic, and intergenic regions, and a very strong depletion in coding exons, although ATAC-seq peaks are slightly more biased towards distal regions relative to promoter peaks than FAIRE-seq (S1 Fig.).

**Fig 3:**
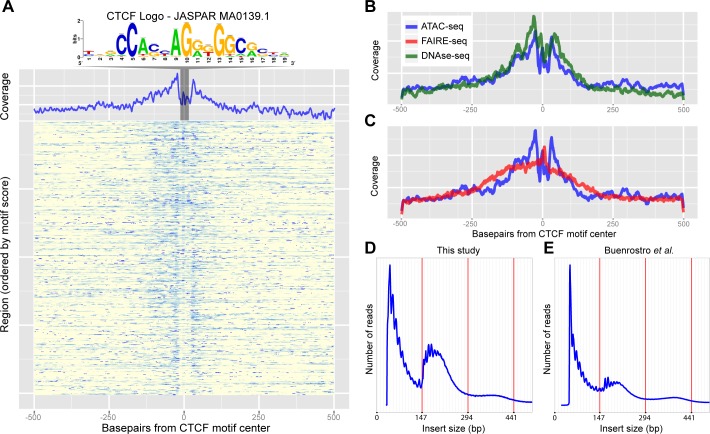
Recovery of known enhancers and CTCF footprinting by ATAC-seq. (A) Heatmap of ATAC-seq Tn5 transposase cut site occurrence in 500bp around 805 CTCF ChIP peaks centered on the JASPAR-MA0139.1 (CTCF) motif, highlighted and shown above. An aggregation plot shows the overall profile and protection at the motif. (B-C) Comparisons of the DNaseI-seq and FAIRE-seq cut sites respectively with the ATAC-seq profile. DNaseI-seq has a very similar profile whilst FAIRE-seq shows enrichment around the motif but with no protection. (D-E) Histograms showing the frequency of insert sizes from both this study in Drosophila (D) and the study by Buenrostro *et al*. in humans (E) [24]. Red lines indicate the amount of DNA bound by one, two and three nucleosomes.

To investigate how accurately these methods can identify promoters, enhancers, and insulators, we looked in turn to each of these three classes of regulatory elements. Firstly, the accessibility of promoters and transcription start sites is overall highly enriched for both methods, although ATAC-seq shows slightly lower background levels, indicating a higher signal-to-noise ratio for ATAC-seq (Fig. 2E). Secondly, to evaluate whether distal open chromatin peaks also identify functional enhancers, we made use of two collections of Drosophila enhancers, namely the REDFly database and the FlyLight [21] database. Out of 56 REDFly enhancers that are active in the eye, ATAC-seq and FAIRE-seq recover 28 and 24 respectively whilst combined they recover 34. The recovery rates for eye enhancers are the highest of all tested enhancer categories from REDFly, which illustrates the specificity of these approaches at similar thresholds in the ranked list of all genomic regions (S2 Fig.). Likewise, eye enhancers from the FlyLight database show increased chromatin activity (Fig. 2F); and the highest overlap between our open chromatin peaks and all FlyLight enhancers is found for eye enhancers (402 out of 576 eye enhancers have an open chromatin peak), much more than for adult brain enhancers and embryo enhancers [22] (Fig. 2G). Although both approaches have a good performance for enhancer detection, ATAC-seq shows a higher recall of true enhancers than FAIRE-seq, detecting ∼18.75% more enhancers (blue versus red bars in Fig. 2G), even at similar levels of specificity (S2 Fig.). Thirdly, we also asked whether the open chromatin peaks overlap with one more regulatory genomic feature, namely functional insulators. To identify insulators, we performed ChIP-seq against CTCF in the wild type eye-antennal disc, under the same conditions as the ATAC-seq and FAIRE-seq (see Materials and Methods). We identified 3682 CTCF peaks, which are significantly enriched for the CTCF motif (PeakMotif adj. p-value = 2.63*10^–15^) (Fig. 3A). Based on this motif, we selected 805 high-confidence CTCF binding sites, having both a significant peak and a significant motif. Both ATAC-seq and FAIRE-seq signals are strongly enriched in the regions around the CTCF binding sites (Fig. 3A-C). Generally, CTCF peaks were identified as accessible regions, with 2244 of all the 3682 CTCF peaks overlapping (minimum 40%) with ATAC or FAIRE peaks. Of the 805 high-confidence CTCF sites, 279 and 278 are effectively (minimum 40% overlap) called as peaks by ATAC-seq and FAIRE-seq respectively, indicating a highly similar detection rate of both techniques. Thus, both approaches allow for efficient genome-wide detection of promoters and enhancers whilst also detecting insulator sites, starting from low input tissue samples.

### TF footprinting and nucleosome packing can be revealed by ATAC-seq

To further analyze the resolution of both open chromatin profiling methods we assessed whether ATAC-seq and FAIRE-seq can be used for transcription factor footprinting [23]. In the original publication of ATAC-seq [10], it was shown that CTCF binding regions, determined by ChIP-seq, show increased ATAC-seq signals, while certain nucleotides within the actual CTCF binding site are protected from tagmentation, similar to protection from DNaseI cleavage in DNaseI-seq. To test whether this is also the case in Drosophila eye development, we centered the 805 high-confidence CTCF peaks on the best scoring occurrence of the CTCF motif (we used the strongest enriched PWM) to investigate open chromatin signals around CTCF binding sites (Fig. 3A) with background signal removed. Similarly to DNaseI-seq, for ATAC-seq the actual CTCF sites show a clear drop in cut sites. This indicates that, although the regions are generally open and therefore accessible by the transposase, the CTCF protein protects its binding site from being cut at specific nucleotides (Fig. 3B-C). This further demonstrates the high degree of resolution obtained with ATAC-seq while identifying active genomic regulatory elements.

A second structural feature of ATAC-seq, as shown in the original publication for human chromatin in vitro, is its ability to correlate distances between cut sites with nucleosomal positioning [24]. To assess whether such information could also be obtained from small in vivo samples in Drosophila, we sequenced two samples with paired-end sequencing at low coverage, and found a distribution of insert sizes that almost perfectly resembles that of human chromatin (Fig. 3D-E). Interestingly, this analysis also shows that even by shallow sequencing, with as few as 0.5 million mapped reads, regulatory regions can be accurately identified across the entire genome due to the high signal-to-noise ratio of ATAC-seq (S3 Fig.), and that both single-end and paired-end sequencing provide near identical results (S4 Fig.). Taken together, these two experiments illustrate that ATAC-seq can identify nucleosome-depleted regions, and protected nucleotides at high resolution, even from low-input in vivo material from a heterogeneous tissue.

### Ras-driven oncogenesis drastically reshapes the open chromatin landscape

**Fig 4:**
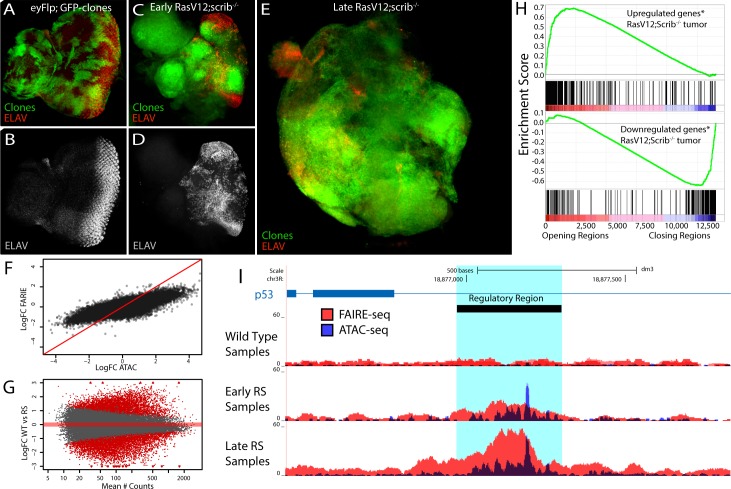
The tumor chromatin landscape. (A-E) Confocal images of eye imaginal discs, clones are indicated by GFP expression (green) and neuronal differentiation is visualized by an ELAV staining (red or grey). (A-B) Control disc showing a normal photoreceptor differentiation pattern (y,w,eyFlp; act>y+>Gal4, UAS-GFP; FRT82). (C-D) Early oncogenesis (day7), the clones are homozygous *scribble* mutant and overexpress Ras^v12^ (Ras^V12^; *scrib* ^-/-^), changing the morphology of the disc and blocking photoreceptor differentiation (y,w,eyFlp; act>y+>Gal4, UAS-GFP, UAS-Ras^V12^; FRT82 scrib^2-^,e). (E) Late oncogenesis (day 9), the clones have overgrown neighboring tissue and formed a massive tumor. (F) Comparing the log fold change of the differential peaks between tumor and wild type, for FAIRE (y-axis) and ATAC (x-axis) shows the greater dynamic range of ATAC. (G) MA-plot of the 36506 peaks with their mean number of counts (x-axis) and their log fold change between wild type and Ras^V12^; *scrib* ^-/-^ (y-axis). 11516 peaks (red) are called significantly different (padj < 0.01) by DESeq2 using the batch effect (see Materials and Methods). (H) Gene Set Enrichment Analysis plots showing a strong correlation between genes [69] up regulated in Ras^V12^; *scrib* ^-/-^ tumors and the opening of nearby regulatory regions (top) and vice versa (bottom). (I) Example of a gradually opening regulatory region in an intron of p53, a known tumor suppressor gene with significantly increased expression in these tumors (logFC = 0.73, padj < 0.0017).

Encouraged by the power of ATAC-seq and FAIRE-seq to identify active regulatory regions in heterogeneous wild type tissues, we combined both methods to map all the differentially activated regulatory regions during tumor development. To this end, we used a well-established cancer model in which the eye disc is transformed by over-expression of oncogenic Ras protein (Ras^V12^) in combination with a homozygous *scrib* ^-/-^ mutation (Fig. 4A-E). The combination of these two perturbations in clones of cells in the eye disc has been shown to generate invasive tumors and to serve as a *bona fide* cancer model [14,25–29], but there is only limited molecular and pathway characterization of these tumors.

To identify which parts of the genome are differentially open in the tumors we performed ATAC-seq and FAIRE-seq both on early tumors (RSE, Fig. 4C,D) and late tumors (RSL, Fig. 4E) (see Materials and Methods) and compared their open chromatin landscapes to that of the wild type tissue. We found that the regulatory landscape in tumors is drastically different from the wild type, having both thousands of significantly increased peaks (4851 for ATAC-seq) and thousands of significantly decreased peaks (4984 for ATAC-seq). Notably, the dynamic range of ATAC-seq seems to be greater than that of FAIRE-seq, as ATAC can detect both smaller and greater significant differences between normal and tumor states than FAIRE (Fig. 4F). Interestingly, when we apply a statistical model allowing for the analysis of both ATAC-seq and FAIRE-seq signals together, the total amount of significantly opening and closing peaks increases to 11516 (Fig. 4G and Materials and Methods); these differential peaks have slightly different genomic distributions, although at present it is unclear which mechanism could be causing this (S5 Fig.).

Since a database with regulatory regions specific for these tumors does not exist, we needed an alternative approach to test whether our candidate regulatory regions are indeed functional regions affecting gene expression of their candidate target genes. To investigate this, we ranked all our regions according to their fold-change between wild type and tumor tissue, and linked them to their candidate target genes based on their location in the genome (see Materials and Methods). Using publicly available gene expression data obtained under the same conditions as ours (GEO accession: GSE42938) [30] we examined the enrichment of the differentially expressed genes in the Ras^V12^; *scrib* ^-/-^ tumors relative to this ranked region-gene list. The enrichment plots in Fig. 4H (by Gene Set Enrichment Analysis, see Materials and Methods) show that the up-regulated genes strongly correlate (p-value < 10^–7^, and Normalized Enrichment Score (NES) = 2.4) with the differentially opening regions, while genes down-regulated in the tumor strongly correlate (p-value < 10^–7^, NES = -2.4) with the genomic regions showing a decrease in open chromatin (differential gene-region pairs are available in S1–S2 Tables). The correlation still holds when we stratify our putative regulatory regions in two groups based on their distance from a TSS (proximal versus distal) or when we assign an alternative way to assign peaks to genes (S6 Fig.). This indicates that the differentially opening/closing chromatin regions in the tumor tissue are overall functional and play a role in the perturbation of the transcriptome. An example of a tumor-specific regulatory region is a previously unknown putative enhancer within an intron of p53 for which the strongly increased chromatin opening likely points to the underlying activation of p53 in the tumor cells (Fig. 4I).

Next, we wanted to test whether the newly opened chromatin during tumor formation corresponds to functional regulatory regions that have an endogenous role in other tissues during development. To this end we took the 4111 differential peaks whose normalized number of reads was at least two fold increased in the tumor samples when compared to the wild type control, and clustered these peaks into 3778 unique candidate regulatory regions that gain activity in the tumor (see Materials and Methods). We compared this set of differentially active regions against the entire collection of REDFly, FlyLight, and VDRC enhancer resources, covering a total of more than 16000 enhancers with at least one tissue of activity. The most significant overlap was found with sets of enhancers known to be active in the “leading edge of invading tissue”, and with “epidermis” and “midgut primordium” (S3 Table). These relationships may indicate re-activation of endogenous invasive processes. Activated regulatory regions also overlap with genitalia enhancers, which may indicate a re-activation of germline expression typical for pluripotent stem cells, corresponding to previous studies [31]. An example of such a gene is *fruitless* (*fru*), for which our data pinpoints a previously described genitalia enhancer [21] that may underlie the observed over-expression of *fru* during the oncogenic process in the Ras^V12^; *scrib* ^-/-^ cancer model, and in another cancer model in the Drosophila brain [31] (S7 Fig.).

In conclusion, profound changes in the open chromatin landscape can be identified between wild type tissue and Ras^V12^; *scrib* ^-/-^ tumor tissue using ATAC-seq and FAIRE-seq. In the tumor, many endogenous enhancers and promoters are ectopically activated, and their activity strongly correlates with changes in gene expression.

### Motif inference on the opening regulatory regions predicts AP-1 and Stat92E as key regulators

**Fig 5:**
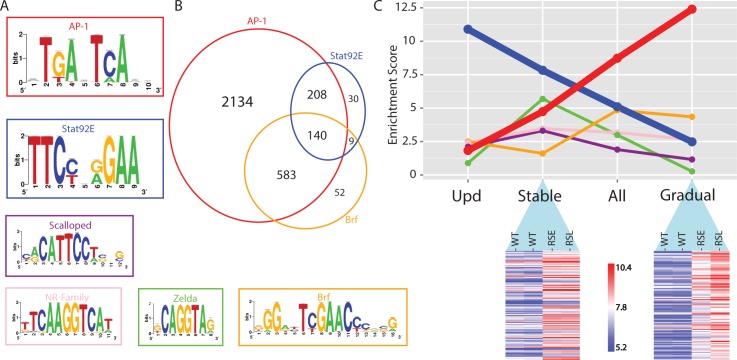
Regulator detection by motif inference. (A) Motifs enriched in the activity-gaining enhancers for the transcription factors AP-1, Stat92E, Scalloped, nuclear receptor family, Zelda and Brf. (B) Venn diagram of the tumor-specific enhancers that are predicted to be responsive to AP-1, Stat92E and Brf transcription factors. (C) The motif enrichment score (i-cisTarget) of the six transcription factors plotted for each of the four different enhancer groups; opening after Upd-overexpression (activating Stat92E), Stably opening enhancers, All opening enhancers and Gradually opening enhancers in Ras^V12^;Scrib^-/-^ tumors. The heatmaps are representing a subset of the regions that are classified as stably or gradually opening. A variance stabilizing transformation (DESeq2) was performed on the count data, blue indicates low and red indicates high peak intensity.

Having identified the exact locations of activity gaining regulatory regions in the tumors downstream of oncogenic Ras^V12^; *scrib* ^-/-^ provides us with a high-quality set of sequences that are likely regulated/bound by a shared set of transcription factors. To predict which transcription factors might be binding to these activity gaining tumor-induced regulatory regions, we used a recently developed motif discovery method, known as i-cisTarget [32]. From a total of 9713 candidate TF motifs (as position weight matrices (PWM)), i-cisTarget yielded the AP-1 and Stat92E motifs as the two most enriched, AP-1 is ranked 1st with a normalized enrichment score (NES) of 8.73 and Stat92E is ranked 2nd with a NES of 5.1. In the active, but unchanging, regulatory regions the Stat92E motif is not enriched and there is only a minor enrichment for the AP-1 motif (NES = 2.7), indicating that the enrichment is specifically in the activity gaining regulatory regions. (see Materials and Methods for an explanation of the NES score). Remarkably, of the tumor-induced regulatory regions, AP-1 is predicted to target the majority (3065 of the 3778, or 81%), pointing to its driving role in oncogenesis. The AP-1 complex is a homo- or heterodimer of bZIP proteins such as Jun and Fos, binds to highly similar DNA motifs, and is functionally activated downstream of phosphorylated Jun Kinases (JNK). Interestingly, multiple labs have shown the importance of JNK signaling, and of the AP-1 complex, specifically to the development of Ras^V12^; *scrib* ^-/-^ tumors. Particularly, either suppressing JNK signaling or knocking-down the AP-1 complex, is sufficient to block tumor development [14,29,33,34]. The Stat92E motif is enriched in a smaller part of the regulatory regions and shows a significant overlap with the predicted AP-1 responsive regions (Fig. 5A, B). This is consistent with reports that show that these pathways can have synergistic effects on Ras^V12^; *scrib* ^-/-^ tumorigenesis [16].

Besides AP-1 and Stat92E, a few additional motifs for other TFs are also significantly enriched, although with a lower representation (Fig. 5A). One of them is the Scalloped (Sd) motif, a transcription factor that acts together with its coactivator Yorkie (Yki) to promote tissue overgrowth, as effectors of the Hippo signaling pathway [35]. It has been shown that knockdown of either Scalloped or Yorkie can rescue *scrib* ^-/-^ mutant tissue overgrowth and reduces Ras^V12^; *scrib* ^-/-^ tumor size [36]. Another group are the zinc-finger protein motifs of Zelda (Vfl) that are known for their activating roles in early *Drosophila* development [37], and for which a role during oncogenesis has not been described. Note that not all enriched motifs are necessarily involved in the regulatory oncogenic program, and some can be “bystander” motifs for the key regulators. An example of such a bystander motif could be the Zelda motif, which often co-occurs with Stat92E motifs in the same regulatory region because these two TFs cooperate during early embryonic development [38], but in the eye tumor the *zelda* gene is not mis-regulated, while all 3 the ligands of the JAK/STAT signaling pathway are (S1 Table). Finally, we recovered several nuclear receptor motifs (e.g., Ftz-f1 and Hr39) and motifs of the TFIIB-related factor (Brf), which increases RNA polymerase III-mediated transcription, and its overexpression has been linked to several human cancer types [39]. Interestingly, this set of candidate Brf-regulated tRNA genes could be discovered by open chromatin profiling, but not by microarrays or mRNA-seq, showing another advantage, and complementarity, of using chromatin profiling besides classical gene expression profiling.

Next we asked whether the activated regulatory regions in the early tumors become even more activated in the late tumors, or if different regulatory regions become activated during tumor progression. In the early tumor, about half of the eye disc still consists of normal tissue, while in the late phase the tumor tissue has overtaken the entire eye disc and invades into the optic lobes of the brain and the ventral nerve cord to a greater extent. By comparing wild type versus early tumors, and early tumors versus late tumors, we found that the majority of changes are gradual, showing an increase in the height of the open chromatin peak between wild type and early tumor, and a further increase between the early and the late tumor. Such a gradual increase of the peak height on a regulatory region most likely indicates that this region is becoming accessible in a higher fraction of cells in the dissected tumor tissue, which is likely the consequence of a lower percentage of normal cells in the late tumor tissue. On the other hand, we found a subset of regulatory regions that are more open in the early tumor versus wild type, but do not show an increasing signal in the late tumor (determined by Fisher’s omnibus, see Materials and Methods). Interestingly, the motif enrichment scores for AP-1 and Stat92E are very different between the gradually and stably opening regulatory regions. More specifically, the stably open regions are mainly enriched for Stat92E (ranked 1st, NES = 7.83), while the enrichment for AP-1 motifs is reduced (ranked 3rd, NES = 4.74). On the other hand, the gradually open regions are strongly enriched for AP-1 motifs (ranked 1st, NES = 12.4), while the Stat92E motif is no longer enriched in this set (NES < 2.5) (Fig. 5C). This finding may indicate that, either Stat92E targets are activated earlier than AP-1 targets, or that a relatively small proportion of cells in the invasive tumor retain Stat92E activity. It could also indicate that Stat92E is active in both tumor and non-tumor cells, and that the secretion of Unpaired ligands from the tumor cells can cause non-autonomous activation of the JAK/STAT pathway in surrounding non-tumor tissue [40].

In conclusion, motif inference on differentially open chromatin peaks during tumor development provides valuable hypotheses about the identity of the transcription factors that are driving the oncogenic regulatory program. Our data confirms previous observations of an important role for AP-1 and Stat92E downstream of Ras^V12^; *scrib* ^-/-^ induced oncogenesis, and now suggests that these two regulators can explain a very large fraction of the changing chromatin landscape.

### Stat92E plays a key role in the oncogenic process

**Fig 6:**
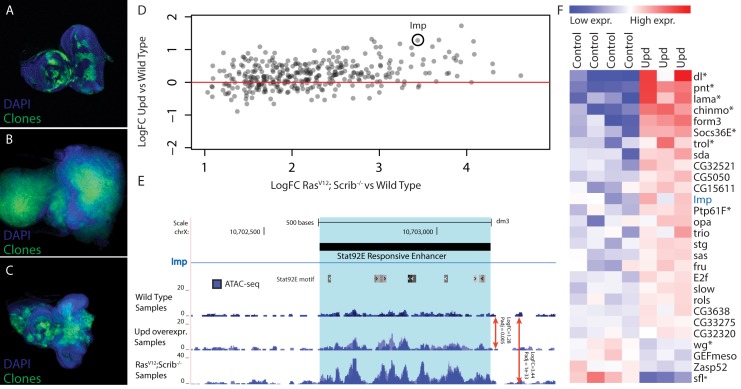
Validation of Stat92E as a key regulator during Ras^V12^; *scrib* ^-/-^ oncogenesis. (A-C) Confocal images of eye-antennal imaginal discs, clones are indicated in green and DAPI in blue. (A) Wild type disc from a 5 day old larva with MARCM clones expressing GFP (B) An eye-antennal tumor taken from an 8 day old larva with the Ras^V12^; *scrib* ^-/-^ clones. (C) An eye-antennal disc from an 8 day old larva with the Ras^V12^; Stat92E^85c9^, *scrib* ^-/-^ clones, showing partial rescue of the tumor phenotype. (D) Opening of predicted Stat92E enhancers from Ras^V12^ *; scrib* ^*-/-*^ tumors vs wild type (x-axis), compared to the opening of these enhancers when ectopically activating the JAK/STAT signaling pathway vs wild type (y-axis). (E) Predicted Stat92E enhancer region inside an intron of *Imp*, the enhancer is significantly more active in the Upd eye discs compared to wild type (logFC 1.28 padj 0.005) and even more so in the Ras^V12^; *scrib* ^*-/-*^ tumors compared to wild type (logFC 3.44 padj 1e-33). (F) Heatmap showing the expression level of 28 genes [42] that have at least one activated enhancer predicted to be Stat92E responsive (Fig. 6A).

One of the predicted transcription factors involved in changing the open chromatin landscape in the tumors is Stat92E, the effector of the JAK/STAT signaling pathway [41]. All three the ligands of this pathway are present in our list of significantly up-regulated gene-peak pairs (S1 Table), strongly supporting the predicted role of JAK/STAT signaling in the tumor. Indeed, previous reports have shown that blocking the activity of the JAK/STAT pathway, for example by using a dominant negative form of the receptor Domeless, reduced the tumor phenotype [16]. However, the direct involvement of Stat92E in this process has not been explicitly tested. We therefore incorporated a null mutation of *Stat92E* in the Ras^V12^; *scrib* ^-/-^ tumors (Stat92E[85c9], see Materials and Methods) to determine if Stat activity is required for the tumor phenotype. We observed that tumor growth was severely reduced (Fig. 6A-C). In addition, in the Stat92E loss-of-function, a fraction of larvae with reduced tumors now also reach pupation stages, which is not observed in Ras^V12^; *scrib* ^-/-^ larvae.

Based on our motif predictions (see above), Stat92E may activate more than three hundred regulatory regions and thereby play a role in regulating the nearby target genes, downstream of Ras^V12^; *scrib* ^-/-^ during oncogenesis. If these promoters and putative enhancers depend on Stat92E for their opening, the same regions may be activated and opened by Stat92E alone, independently of the Ras^V12^; *scrib* ^-/-^ induced oncogenesis. To test this we hyper-activated the JAK/STAT pathway in the wild type eye disc, and thereby the downstream Stat92E activity, by overexpressing one of the ligands that is up-regulated in the tumor, namely Unpaired (Upd, encoded by *os*). ATAC-seq on these discs could demonstrate that the tumor-induced changes at Stat92E predicted target sites are recapitulated by JAK/STAT activation alone. The changes are significant and mainly in the same direction (Fig. 6D), but as expected the changes in open chromatin caused by the Upd overexpression are quantitatively more subtle compared to those in the tumors. For example, an intronic regulatory region of *Imp* shows activated chromatin by Stat92E (log2 fold change = 1.23) but this activation is stronger during Ras^V12^; *scrib* ^-/-^ tumor formation (log2 fold change = 3.44) (Fig. 6E).

In the Ras^V12^; *scrib* ^-/-^ tumors we had identified 356 candidate target regulatory regions of Stat92E, of these we found that 72% have a positive fold change in the Upd over-expressing discs (Fig. 6D), indicating that this group is Stat92E responsive (p-value 0.0097). However, this still means that 28% of the candidate regions did not respond to the activation of Stat92E by overexpressing Upd. We analyzed these responsive and non-responsive regions and found that the AP-1 motif is only enriched in the regions that are not responsive to Stat92E, indicating that for those regions AP-1 might be the main input and the Stat92E motif might be of less importance here.

Next, we asked whether these changes in *cis*-regulatory activity directly affect gene expression of target genes. To test this, we linked the 254 Stat92E responsive regulatory regions to nearby (<5kb upstream or intronic) candidate target genes and examined their expression in Upd over-expressing discs using publicly available gene expression data (GEO accession: GSE15868) [42]. After applying two filtering steps, requiring the regulatory region to go significantly open both in tumor and UPD-overexpressing tissue, combined with a significant differential expression of the assigned gene, we end up with a final list of 28 potential direct Stat92E target genes (Fig. 6F). Among these are at least seven well-known transcriptional targets of the JAK/STAT signaling pathway including: *dorsal*, *pointed*, *lama*, *chinmo* and *trol*, as well as two key negative regulators of this pathway: *Socs36E* and *Ptp61F* [42–49]. Interestingly, not all genes that have an opened Stat92E regulatory region are up-regulated. The JAK/STAT pathway can also repress transcription of target genes and is known to block the Wnt/Wingless signaling pathway in the eye imaginal disc [50]. In our list we recover two genes that are known to be involved in the Wingless signaling pathway, *wingless* itself and *sulfateless*, an essential enzyme for this pathway [51]. This may indicate that Stat92E can function directly in the repression of genes, through an as yet unidentified mechanism. An interesting future challenge will be to understand the *cis*-regulatory mechanisms and possible co-factors that determine the mechanisms by which Stat92E can act as activator or repressor.

In conclusion, we demonstrate that Stat92E is a significant transcriptional regulator and required for the growth of our tumor model. We identify known and newly predicted Stat92E targets in the tumor and are able to independently recover >70% of those targets using a Stat92E activation model in the normal eye. This second analysis also illustrates the feasibility of an integrated approach of ATAC-seq and motif discovery to capture and annotate modest, yet functional, changes in the chromatin landscape. We conclude that Stat92E induced chromatin-opening correlates with a change in the transcription of nearby genes, and that Stat92E may function as a key regulator of the tumor transcriptome.

## Discussion

Genome-wide characterization of all promoters and enhancers controlling a particular gene expression profile and/or phenotype is a key challenge for understanding the regulatory underpinnings of any biological process in vivo. Our study first compares, and subsequently combines, two recent methods for open chromatin profiling to obtain genome-wide regulatory landscapes in the developing Drosophila eye as model system. By applying FAIRE-seq and ATAC-seq to the normal eye-antennal imaginal disc, alongside anti-CTCF ChIP-seq, we found both methods to be highly robust in identifying accessible or open regions, with few differences between strains with different genetic backgrounds. The main advantages of ATAC-seq, for our application, are besides its undemanding experimental procedure, (1) its higher signal-to-noise ratio, with low background signal and sharper peaks; (2) its ability to identify TF footprints, as binding sites are protected from transposon insertion, similar to their protection from DNaseI cleavage [23]; and (3) its ability to determine nucleosome positioning when using paired-end sequencing.

Although these assets of ATAC-seq may be important for some studies, overall both FAIRE and ATAC allow identification of promoters and candidate enhancers, and here we use them as independent “replicate” measurements (taking batch effects into account) to examine the open chromatin status of a tumor model.

We observe that the *cis*-regulatory landscape of active promoters and enhancers changes dramatically in Ras^V12^; *scrib* ^-/-^ eye tumors, while more moderate changes were observed in tissues with JAK/STAT induced hyperplasia. A possible explanation for the marked differences in the intensity of change may be found in the cellular composition of the samples. Open chromatin signals represent the average signal across all cells within a sample, and since each regulatory region, in each cell can yield only two open alleles, this observed activity mainly reflects the number of cells in which the region is active, rather than the quantitative activity of the region.

Once the repertoire of tumor-induced regulatory regions was identified by open chromatin profiling, we reasoned that if many of these functional regions are activated by a small set of master regulators, then the motifs of these TFs should be enriched within the sequences of these regions. While motif inference on *gene* sets is challenging due to the large intergenic and intronic regulatory space around genes, motif inference on *enhancer*-size regulatory sequences often gives highly accurate results [32,52–54]. We used the tool i-cisTarget, which is optimized for Drosophila genomes, and found not only AP-1 and Stat92E, but also Zelda, Scalloped (the Hippo pathway effector), Brf, and Ftz-f1 as candidate regulators of the oncogenic, Ras-dependent program. All of these TFs (except perhaps Zelda) can be directly linked to cancer-related processes that are conserved to human [35,39], and AP-1, Stat, and Scalloped have each been previously linked to the Ras^V12^; *scrib* ^-/-^ program specifically [16,29,36,55,56]. We could confirm that the Ras^V12^; *scrib* ^-/-^ tumor phenotype depends on Stat92E, and furthermore reveal that JAK/STAT signaling causes specific chromatin changes at Stat92E-responsive regulatory regions.

Finally, once the regulatory regions and their candidate regulators are identified, an important next step is to examine which target genes are now differently regulated, as a consequence of the activation of these promoters and enhancers. Previous work has found that open chromatin peaks obtained across cell lines are correlated with gene expression changes of “nearby” target genes [4]. We also found evidence that a high degree of chromatin changes are concordant with transcriptional changes of nearby target genes, both in the tumor and in the overgrown tissue with hyper-activated JAK/STAT, confirming that the chromatin state of these regions is not only altered, but that they are also *functionally* activated. Interestingly, not all putative target genes with an increased open chromatin peak are up-regulated, but a small subset is also down-regulated, which indicates that regulatory regions can be “activated” by TF binding and nucleosome depletion, but that the consequence of this activation can also be gene repression (e.g., if the bound TF act as a repressor).

Overall, our integrated approach reveals a large *cistrome* of changing activity at promoters and enhancers during tumor development, which is mainly operated by AP-1 and Stat92E; and illustrates how integrative open chromatin profiling, motif detection, and gene expression analyses have great potential to unravel tissue and cell type specific regulatory programs in vivo, in health and disease.

## Materials and Methods

### Fly stocks

The following fly stocks were used during this investigation: y,w,eyFlp; act>y+>Gal4, UAS-GFP; FRT82 tub Gal80 and y,w; UAS-Ras^V12^; FRT82 scrib^2^, e/ TM6B, yw; UAS-Ras^v12^; FRT82 Stat92E^85c9^, scrib^2^, e/ FRT82 tub-Gal80, Optix-GFP [57] and UAS-GFP:Upd (Courtesy of Fernando Casares), GMR-Gal4, isogenic wild type DGRP-208 (Bloomington stock 25174), y, w; FRT82 and CantonS wild types. Crosses were raised at 25°C on a yeast based medium.

### Dissections

Wild type, Ras^V12^; *scrib* ^-/-^ early and Upd overexpression eye-antennal discs were dissected from wandering third instar larvae (day 6) in 1xPBS. Ras^V12^; *scrib* ^-/-^ late discs were collected three days after larvae began wandering (day 9); this is possible because Ras^V12^; *scrib* ^-/-^ do not pupate, but can persist more than one week in a prolonged larval stage. Immunohistochemistry was performed as previously described in [58]. Confocal images were taken on an Olympus FV1000 or FV1200 microscope. Images were processed using ImageJ and Adobe Photoshop software.

### ATAC-seq

The previously described ATAC-seq protocol was adapted for working with Drosophila rather than human cells [10]. Ten eye antennal imaginal discs (or three Ras^V12^; *scrib* ^-/-^ late total tumors) were immediately placed in 50 μl ice cold ATAC lysis buffer (10 mM Tris-HCl, pH 7.4, 10mM NaCl, 3mM MgCl_2_, 0.1% IGEPAL CA-630). Lysed discs were then centrifuged at 800 xg for 10 minutes at 4’C and the supernatant was discarded. The rest of the ATAC-seq protocol was performed as described previously [10] using the following primers: Fwd:- ‘AATGATACGGCGACCACCGAGATCTA CACTCGTCGGCAGCGTCAGATGTG’ and Rev:- ‘CAAGCAGAAGACGGCATACGAGATXXX XXXGTCTCGTGGGCTCGGAGATGT’ (where X indicates barcode nucleotides). The final library was purified using a Qiagen MinElute kit (Qiagen) and Ampure XP beads (Ampure) (1:1.2 ratio) were used to remove remaining adapters. The final library was first checked on an Agilent Bioanalyzer 2000 for the average fragment size. Resulting successful libraries were sequenced with 50bp, single end reads on the Illumina HiSeq 2000 platform. Single end sequencing was chosen for this study because we were not interested in the fragment contents (i.e., how many nucleosomes are placed between two insertion sites), rather just the profile of insertion sites, which also made comparisons with pre-existing FAIRE-seq data easier.

### FAIRE-seq

Methodology adapted from [12]. In short, 150 head complexes were dissected from wandering third instar larvae, these were fixed for 10 min with 4% formaldehyde,. The formaldehyde was then replaced with 750 μl quenching buffer (125 mM Glycine 0.01% Triton X-100 in PBS) was added and incubated at room temperature for 10 minutes. Quenching buffer was replaced with buffer A (10 mM HEPES-KOH pH8.0, 20 mM EDTA pH8.0, 1mM EGTA pH8.0, 0.25% Triton X-100, 1mM PMSF) and 200 eye-antennal discs were then dissected in buffer A and kept on ice, these were centrifuged at 6000rpm, 4’C to pellet the discs and lysed in lysis buffer 1 (50mM HEPES-KOH, pH 7.5, 140mM NaCl, 1mM EDTA, 10% glycerol, 0.5% NP-40, 0.25% Triton X-100) rocking at 4’C for 10 minutes, centrifuged and the supernatant removed. Next lysis buffer 2 (10mM Tris-HCl, pH 8.0, 200 mM NaCl, 1mM EDTA, 0.5 mM. EGTA) was added and the sample was rocked at room temperature for 10 minutes. Finally lysis buffer 3 (10 mM Tris-HCL, pH 8.0, 100mM NaCl, 1mM EDTA, 0.5mM EGTA, 0.1% Na-deoxycholate, 0.5% N-lauroylsarcosine) was added and samples were sonicated (Bioruptor UCD-200 (Diagenode) at 4’C, 8 Cycles set to pulse high 30 seconds, rest 30 seconds) immediately.

Double phenol/chloroform extraction was performed with a final chloroform extraction. DNA was precipitated using Sodium acetate (0.3 M, pH 5.2), 20mg Glycogen and 100% ethanol. DNA was washed with 500 μl ice cold 70% Ethanol. Supernatant was removed and the pellet air-dried. The dried pellet was re-suspended in 50 μl TE buffer (10 mM Tris pH 8.0, 1 mM EDTA in MilliQ water) and incubated at 65’C overnight to reverse crosslinks. Finally 1 μl 10mg/ml RNAseA was added and incubated at 37’C for 1 hour, samples were cleaned using the QiaQuick MinElute kit (Qiagen) and DNA was measure using a Qubit analyzer. Final libraries were prepared as per standard Illumina protocols.

### ChIP-seq

Head complexes were dissected from wandering third instar larvae (500, in batches of 100) and fixed in 1ml crosslinking solution (1.8% formaldehyde, 50 mM HEPES pH 7.9, 1 mM EDTA, 0.5 mM EGTA, 100 mM NaCl in MilliQ water) for 25 minutes at room temperature while rotating. Crosslinking solution was replaced 5 times after 5 minutes each time. Crosslinking was stopped by adding 1ml stop solution (0.01% Triton X-100, 125 mM Glycine in PBS) and incubating at room temperature for 10 minutes while rotating, this was repeated for 3 more washes. Complexes were washed in 1 ml wash A (10 mM HEPES pH7.9, 10 mM EDTA, 0.5 mM EGTA, 0.25% Triton-X100 in MilliQ water) for 10 minutes at room temperature while rotating. Complexes were washed in 1 ml wash B (10 mM HEPES pH7.9, 200 mM NaCl, 1 mM EDTA, 0.5 mM EGTA, 0.01% Triton X-100 in MilliQ water) for 10 minutes at room temperature while rotating, this was repeated 3 more times. Eye-antennal discs (200) were dissected from head complexes in wash B and collected in a tube containing 100 μl sonication buffer (10 mM HEPES pH7.9, 1mM EDTA, 0.5 mM EGTA in MilliQ water), samples were sonicated (Bioruptor UCD-200 at 4’C, 8 Cycles set to pulse high 30 seconds, rest 30 seconds) immediately. Samples were centrifuged at 21000 xg for 10 minutes at 4’C. For each immunoprecipitation, the pellet from 100 μl extract was re-suspended in 1 ml RIPA buffer (140 mM NaCl, 10 mM Tris-HCl pH8.0, 1 mM EDTA, 1% Triton X-100, 0.1% SDS, 0.1% Na-deoxychoate, 1% PMSF in MilliQ water), an extra sample (10 μl) was kept aside as input.

Immunoprecipitation was performed by adding 20 μl protein A/G magnetic beads (Millipore) and incubating for 1 hour at 4’C; samples were centrifuged at 3000 rpm for 2 minutes and supernatant kept. Anti-CTCF antibody (10 μl crude rabbit serum—A kind gift from Dr. Ranier Renkawitz) was added to the supernatant and rotated at 4’C overnight. Immunocomplexes were recovered by adding 20 μl protein A/G magnetic beads to sample and incubating at 4’C for 3 hours while rotating. Magnetic beads were separated using a magnetic stand and supernatant discarded. Beads were re-suspended and washed for 5 minutes on a rotating platform, with 1 ml of the following buffers in order:- Low salt immune complex wash buffer (Millipore), High salt immune complex wash buffer(Millipore), LiCl immune complex wash buffer (Millipore), TE buffer. ChIP elution buffer (Millipore) was warmed to 38’C and 100 μl was added to the beads, 1μl RNaseA was also added, samples were incubated at 36’C for 30 minutes, shaking at 950rpm. Both immunoprecipitated and control samples had 1ul proteinase K added and were incubated at 62’C for 2 hours, shaking at 950rpm and then 95’C for 10 minutes. Samples were allowed to cool to room temperature and beads were separated with a magnetic stand. Final DNA was purified according to the manufacturers guidelines (MagnaChIP, Millipore) Final libraries were prepared as per standard Illumina protocols.

### ATAC-seq, FAIRE-seq, and DNaseI-seq data analysis

Raw reads were first cleaned for adapter sequences using *fastq-mcf* using default parameters and an adapter file containing common Illumina adapter sequences. Cleaned samples were mapped to the Drosophila melanogaster FlyBase release r5.53 genome [59] using *bowtie2* [60] (default parameters) and reads with a mapping quality of less than 4 were discarded. In several samples we discovered reads mapping to the Wolbachia genome (60–80%), these were also discarded. Both ATAC-seq and DNaseI-seq reads (publicly available data) were adjusted to better represent the open chromatin by centering reads on the cut-sites and extending this by 5bp on either side, samples were finally sorted and indexed using Samtools [61]. Peaks were called on ATAC-seq, FAIRE-seq and DNaseI-seq samples using the MACS2 software suite [62] with the added parameters “-g dm –nomodel –shiftsize 50 –q 0.01”. For comparison between samples, all peaks from each sample were merged to provide one set of combined peaks. Peaks on ChIP-seq data were called also using the MACS2 software suite with the parameters “-g dm –q 0.01” also using input as a control. For quantification of peaks, bed files of combined peaks were converted into a GFF3 format and then the number of reads per peak, per sample were counted using htseq-count [63].

Finally bigWig files were created from bam files for each sample using genomeCoverageBed [64] (using the –scale option) and bedGraphToBigWig [65]. Scales were determined by the ‘sizeFactors()’ command from DESeq2 [66] on a matrix of all samples, counted on all combined peaks. Genomic locations of peaks was determined by the CEAS software package using default parameters and a prebuilt dm3 gene annotation file [67]. For the CTCF protection analysis motif scanning was performed with Cluster Buster to find occurrences of the JASPAR-MA0139.1 motif in CTCF peaks using the parameters ‘-m7 –c0’, each peak was then re-centered on the center of the best scoring motif present. The cut sites from each read was determined and plotted 500bp around the re-centered CTCF peaks. Signal from the same motif at random regions of DNA was subtracted to remove background noise.

To determine overlaps between peaks, the tool intersectBed was used, with all CTCF peaks or high confidence regions as file A and the ATAC-seq/FAIRE-seq peaks as file B, the option ‘-f 0.4’ was also used to enforce a 40% overlap of the CTCF peak by the ATAC-seq/FAIRE-seq peak.

To identify differential peaks between conditions we used DESeq2 [66] with a p-adjusted cutoff of 0.01. This cutoff was supported by the leading edge of a Gene Set Enrichment Analysis (GSEA [68]) analysis whereby all genes ranked by their most significant peak (tumor vs wild type) are compared to a Ras/Scrib gene signature. To link peaks to genes we assigned a peak within any intron or in the 5kb upstream region of the TSS to the gene. To rank genes according to peak heights (for GSEA analysis) we used the peak with the most significant adjusted p-value. When FAIRE-seq and ATAC-seq are used as replicates, we took batch effects into account in DESeq2.

Rankings for the recovery curves seen in S2 Fig. were generated by scoring peaks (ATAC-seq wild type and FAIRE-seq wild type, merged) based on the number of reads falling within the region for the appropriate samples. ATAC-seq and FAIRE-seq were ranked individually and their recoveries overlaid.

### Publicly available microarray data

We acquired two Affymetrix Drosophila Genome 2.0 Array data sets from GEO [30], one comprised of 3 wild type and 3 Ras^V12^; *scrib* ^-/-^ biological replicates (GEO accession: GSE42938) [69], and the second comprised of 5 wild type and 5 Upd overexpression biological replicates (GEO accession: GSE15868) [42]. We discarded three low-quality samples from our analyses (GSM398336, GSM398339, GSM398341). Differential expression analysis was carried out in R using the Bioconductor packages affy, limma, Biobase and GEOquery, applying a standard limma protocol [70]. After obtaining differential values we associated each probe to their respective gene; for genes with more than one associated probe, we decided to use the probe with the most significant adjusted p-value.

### Gene set enrichment analysis

The differential peaks (wild type control vs Ras^V12^; *scrib* ^-/-^ tumors) were assigned to genes (<5kb upstream or intronic) and for each gene the most significantly differential peak was kept. The genes were ordered based on the relative openness of their assigned peak (opening on top and closing on bottom), to obtain the ranked gene list (x-axis of GSEA plots).

Two groups of differentially expressed genes were determined based on the publically available micro array data (GEO accession: GSE42938). One group contained the significantly upregulated and the other the significantly downregulated genes in the tumors. We used GSEA (with 100000 perturbations) to determine if these groups of differentially regulated genes were significant enriched on either side of the ranked gene list.

### Grouping regulatory regions

The globally opening regions in the Ras^V12^; *scrib* ^-/-^ tumors were determined by differential peak calling between all 5 wild type controls and all 4 Ras^V12^; *scrib* ^-/-^ samples (combining early and late). Of the significantly differential peaks, only those with a logFC greater than 1 were selected, ending up with 4111 globally opening regions.

To determine the early versus late Ras^V12^; *scrib* ^-/-^ regulatory regions, differential peaks were called between the 5 wild type controls and the 2 Ras^V12^; *scrib* ^-/-^ early samples and between the 2 Ras^V12^; *scrib* ^-/-^ early samples and the 2 Ras^V12^; *scrib* ^-/-^ late samples. We selected a subset of regulatory regions that were opening from WT to RSE (with a logFC > 1) and that remained at similar levels between RSE and RSL (-0.2 < logFC < 0.2); we defined these regulatory regions as ‘stably opening’. For the regulatory regions defined as ‘gradually opening’, we selected the regions that are becoming more open between WT and RSE (logFC > 0) and that further open between RSE and RSL (logFC > 0.5). Using a Fisher's Omnibus test we combined the p-values for each regulatory region (one from WT vs RSE and one from RSE vs RSL) and obtain a new chi-squared p-value.

The regulatory regions opening in Upd-overexpression were determined by differential peak calling between the 3 ATAC-seq wild type controls and the 2 ATAC-seq Upd-overexpressing samples. We took the top 250 opening regions (ordered on singed P Value) to perform motif enrichment analysis.

### Motif enrichment and normalized enrichment scores

For motif enrichment we used i-cisTarget [32], a tool developed in our lab to discover motifs significantly enriched in our four regulatory region groups (the stably, globally and gradually opening in Ras^V12^; *scrib* ^-/-^ and opening in Upd-overexpression). We ran i-cisTarget via the command line with the rank threshold = 10000, enrichment score threshold = 2 and a collection of 9713 motifs. The enrichment of each motif in the input set is calculated as an area under the recovery curve (AUC), whereby recovery is observed over a genome-wide ranking of 136K a priori defined candidate regulatory regions [32]. The AUC score is normalized by subtracting the mean of all AUCs over all motifs, and dividing it by the standard deviation, to obtain a Normalized Enrichment Score (NES). We use a cutoff of NES > 2.5 to select significantly enriched motifs. Relationships between NES and False Discovery Rates can be found in [71]. For each factor of interest with multiple enriched motifs, we selected the motif with the highest NES score.

### Accession numbers

ATAC-seq, ChIP-seq and FAIRE-seq data for all conditions are available in GEO (http://www.ncbi.nlm.nih.gov/geo/), with accession number GSE59078.

### Data access

Genome browser tracks for all data, and called peaks for wild type and cancer-related regulatory regions are all available within a UCSC Genome Browser hub from this URL: http://genome.ucsc.edu/cgi-bin/hgTracks?db=dm3&hubUrl=http://ucsctracks.aertslab.org/ATAC-paper/hub.txt

## Supporting Information

S1 Fig Wild type peak locations. Genomic locations of peaks identified in the wild type samples for both ATAC-seq and FAIRE-seq. Both techniques are clearly enriched for promoter regions and depleted for exonic regions, a pattern expected for regulatory elements.
(TIF)

S2 Fig Recovery curves of curated enhancers. Recovery curves which show how well both ATAC-seq and FAIRE-seq recover overlapping enhancer between their peaks and two sets of curated enhancers (FlyLight eye and REDFly eye) in comparison to all other enhancer groups from FlyLight (Janelia farm), REDFly and VDRC. ATAC-seq shows clear enrichment over all other enhancer groups whilst FAIRE-seq seems to only be slightly enriched for REDFly eye enhancers.
(TIF)

S3 Fig Low read depth paired-end ATAC-seq profiles. ATAC-seq data (Blue) from two samples sequenced paired end on an Illumina MiSeq with a low amount of reads, in comparison to our previous FAIRE-seq (Red) data. The wild type sample (Top) was infected with Wolbachia and had only ∼500k reads and the Ras^V12^;*scrib* ^-/-^ sample (Track #3) had ∼1.4 million read. Each FAIRE-seq sample has ∼10 million reads.
(TIF)

S4 Fig Single- and Paired-end sequencing comparison. ATAC-seq data from both single-end (tracks 1 and 3) and paired-end (tracks 2 and 4) of Wild type and Ras^V12^;*scrib* ^-/-^ samples. Each of the sequencing types shows near identical profiles, with the difference most likely attributable to the difference in sequencing depth (∼1 million reads per paired end sample and 10 million per single end sample).
(TIF)

S5 Fig Differentially open tumor peak genomic locations. Genomic location of the differentially opening regulatory regions of the RS-tumor tissue vs wild type compared to the genome. The opening regions have the strongest enrichment in the introns, while the closing regions show a stronger enrichment in the distal intergenic regions.
(TIF)

S6 Fig GSEA analysis of putative promoter and enhancer regions and of alternative peak to gene assignment. GSEA analysis with the same up- and down regulated gene sets of the RS-tumor as in Fig. 4H. The differential peaks are now separated according to their distance to the closest Transcription Start Site and based on this separation a new ranked gene list was created (see Materials and Methods). (A) Ranking based on promoter proximal peaks within 500bp of TSS and (B) based on the promoter distal peaks outside this 500bp. (C) Ranking based on promoter proximal peaks within 1kb of TSS and (D) based on the promoter distal peaks outside this 1kb. (E) The peak to gene assignment is now purely based on their nearest TSS, this way all peaks are assigned to a gene and for each gene the most significant differential peak is retained to create the ranked gene list.
(TIF)

S7 Fig An enhancer identified in the *fruitless* gene. Regulatory region inside an intron of *fruitless* overlapping with a previously described Janelia genital enhancer (R22G01). The enhancer gains activity in the RS-tumor (4 times more reads) and may underlie the increased transcription of the *fruitless* gene.
(TIF)

S1 Table Genes with their assigned Regulatory Region, both going UP in RS-tumors compared to wild type. (DOCX)

S2 Table Genes with their assigned Regulatory Region, both going DOWN in RS-tumors compared to wild type. (DOCX)

S3 Table Enhancer sets enriched in opening or closing ranked lists of differential regulatory regions from RS-tumors. (DOCX)
