# Supplementary figures and images for: Discovery of Transcription Factors and Regulatory Regions Driving In Vivo Tumor Development by ATAC-seq and FAIRE-seq Open Chromatin Profiling
Source: PLoS Genet. 2015 Feb 13;11(2):e1004994. doi: 10.1371/journal.pgen.1004994 (PMC4334524; doi:10.1371/journal.pgen.1004994)

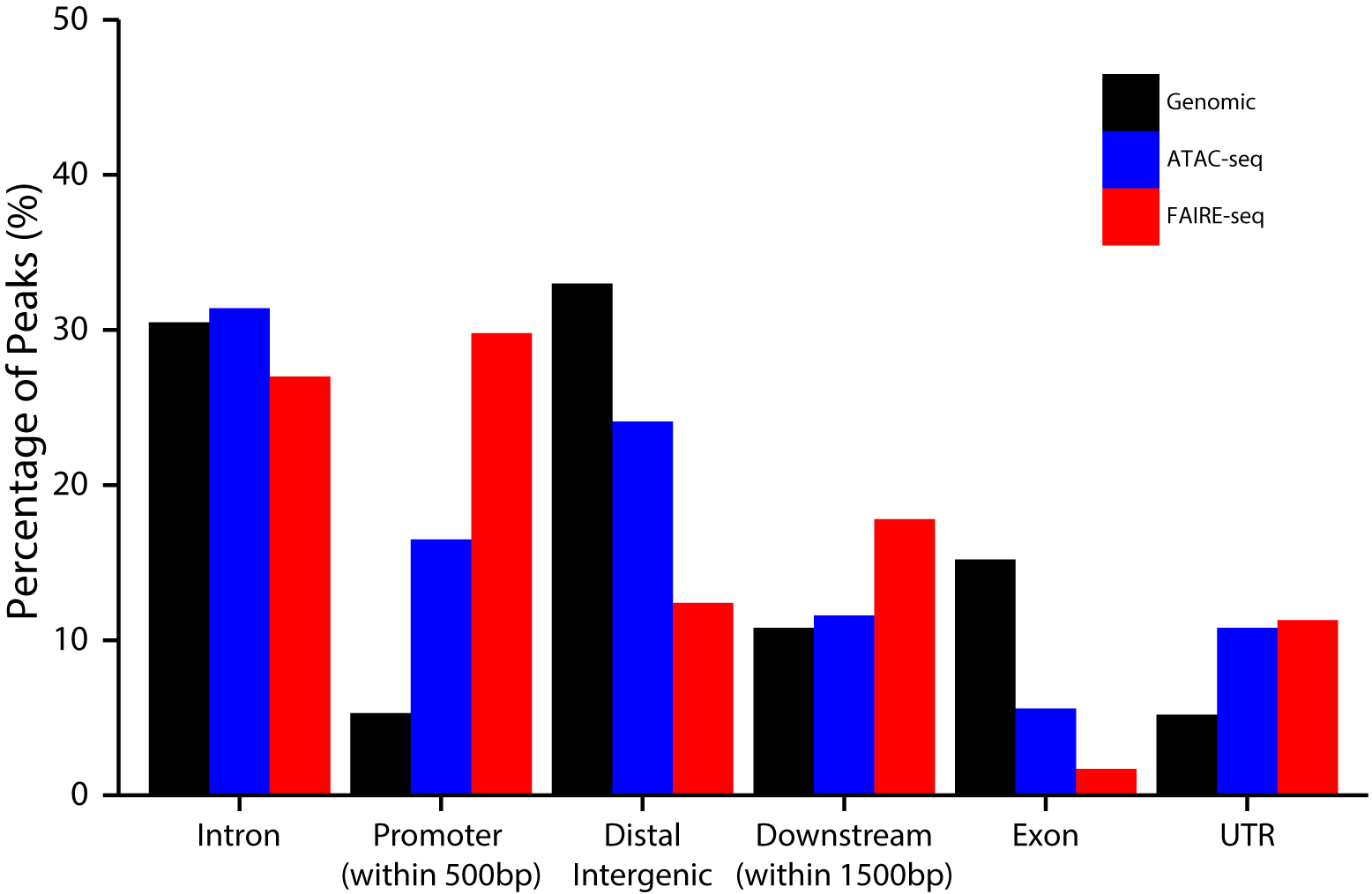

Supplement: S1 Fig — Genomic locations of peaks identified in the wild type samples for both ATAC-seq and FAIRE-seq. Both techniques are clearly enriched for promoter regions and depleted for exonic regions, a pattern expected for regulatory elements. (TIF) [file pgen.1004994.s001.tif]

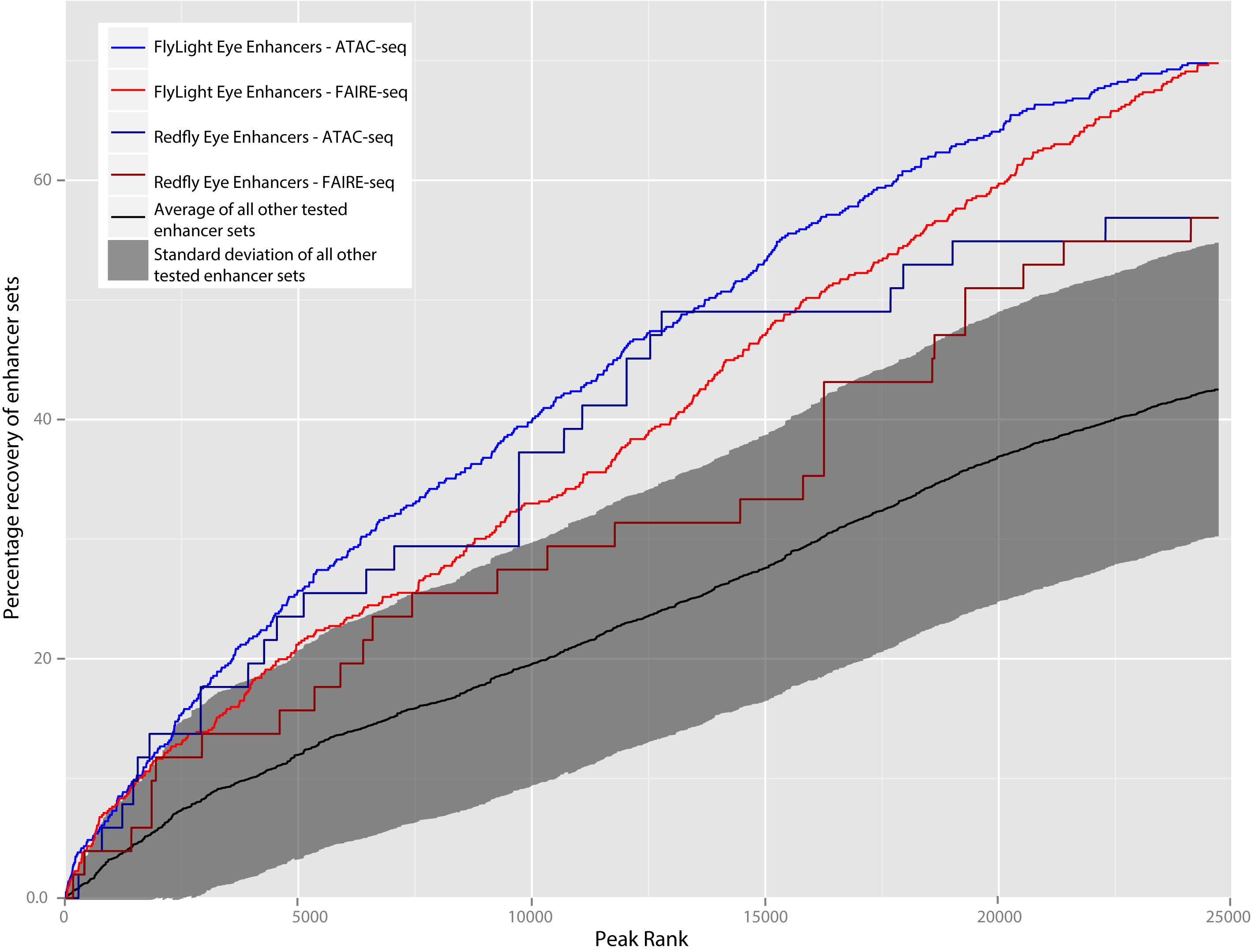

Supplement: S2 Fig — Recovery curves which show how well both ATAC-seq and FAIRE-seq recover overlapping enhancer between their peaks and two sets of curated enhancers (FlyLight eye and REDFly eye) in comparison to all other enhancer groups from FlyLight (Janelia farm), REDFly and VDRC. ATAC-seq shows clear enrichment over all other enhancer groups whilst FAIRE-seq seems to only be slightly enriched for REDFly eye enhancers. (TIF) [file pgen.1004994.s002.tif]

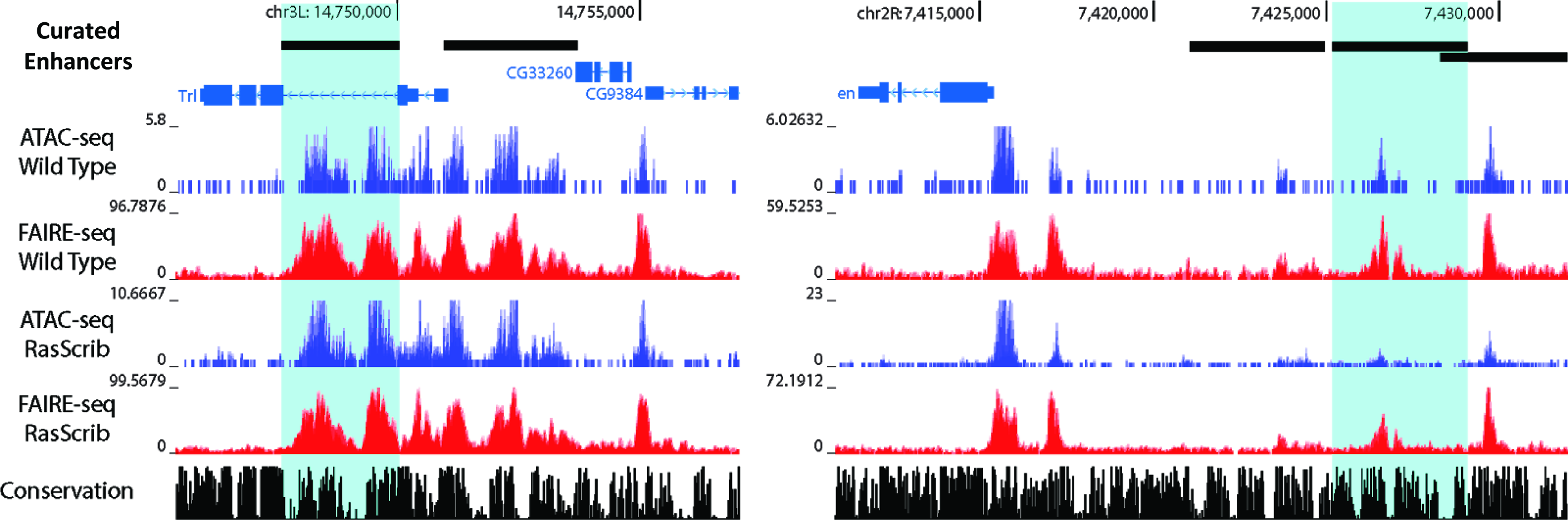

Supplement: S3 Fig — ATAC-seq data (Blue) from two samples sequenced paired end on an Illumina MiSeq with a low amount of reads, in comparison to our previous FAIRE-seq (Red) data. The wild type sample (Top) was infected with Wolbachia and had only ∼500k reads and the RasV12;scrib -/- sample (Track #3) had ∼1.4 million read. Each FAIRE-seq sample has ∼10 million reads. (TIF) [file pgen.1004994.s003.tif]

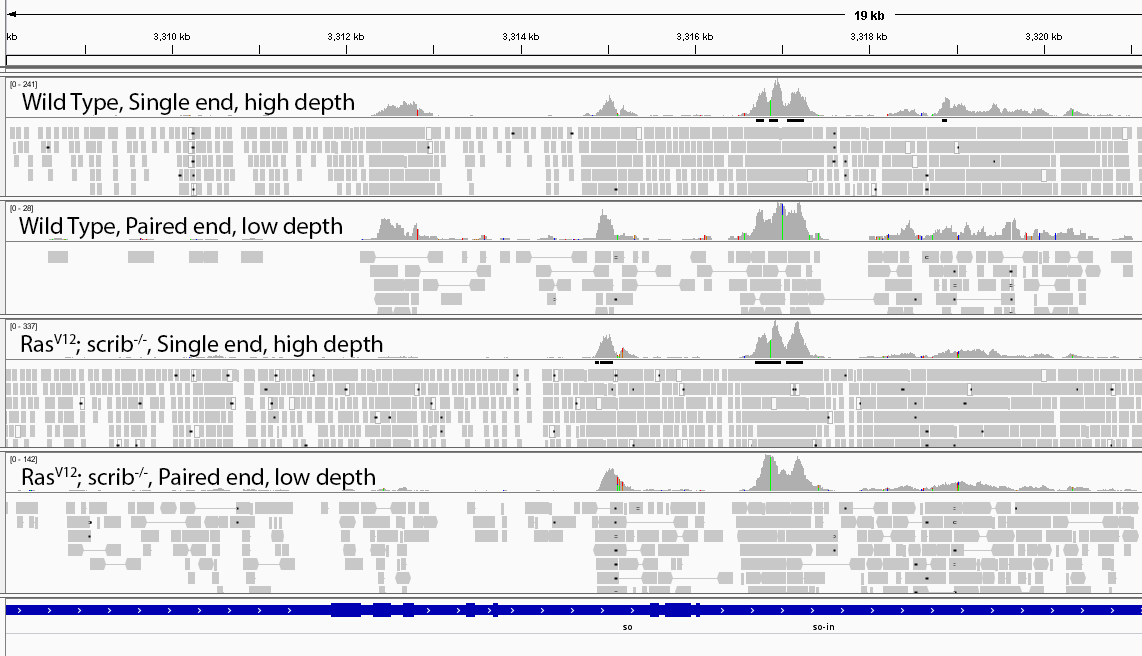

Supplement: S4 Fig — ATAC-seq data from both single-end (tracks 1 and 3) and paired-end (tracks 2 and 4) of Wild type and RasV12;scrib -/- samples. Each of the sequencing types shows near identical profiles, with the difference most likely attributable to the difference in sequencing depth (∼1 million reads per paired end sample and 10 million per single end sample). (TIF) [file pgen.1004994.s004.tif]

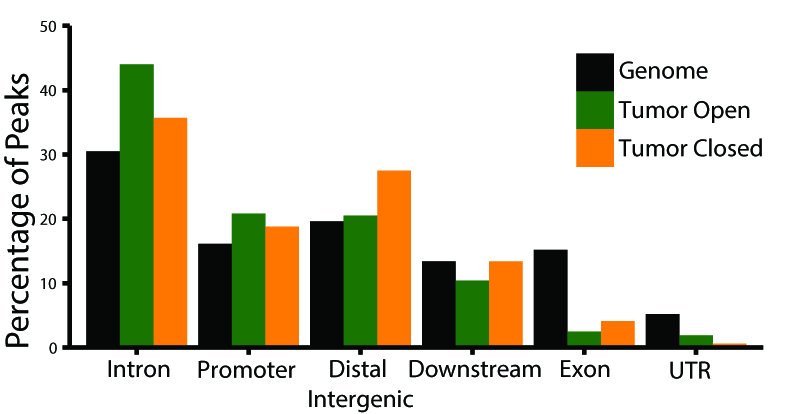

Supplement: S5 Fig — Genomic location of the differentially opening regulatory regions of the RS-tumor tissue vs wild type compared to the genome. The opening regions have the strongest enrichment in the introns, while the closing regions show a stronger enrichment in the distal intergenic regions. (TIF) [file pgen.1004994.s005.tif]

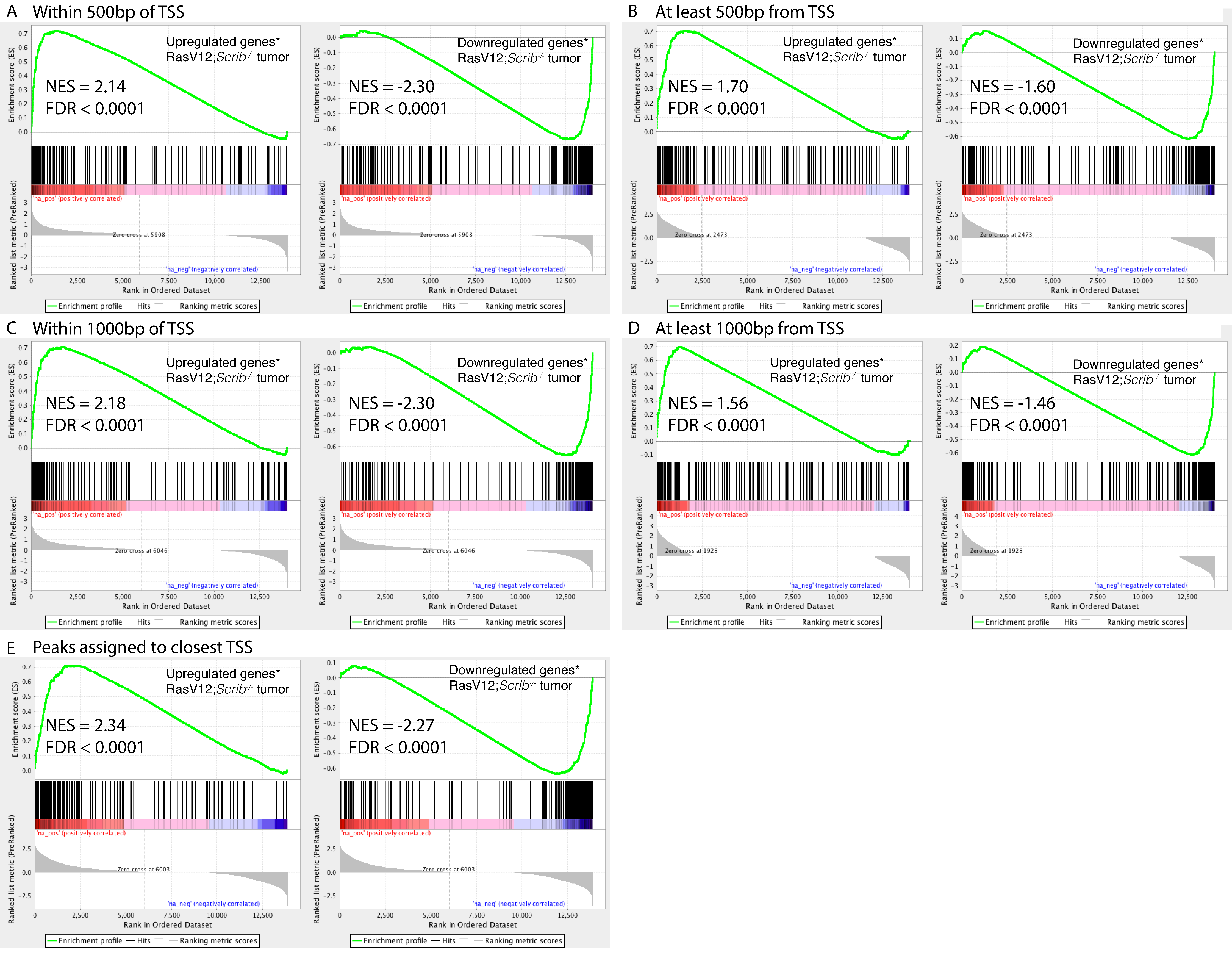

Supplement: S6 Fig — GSEA analysis with the same up- and down regulated gene sets of the RS-tumor as in Fig. 4H. The differential peaks are now separated according to their distance to the closest Transcription Start Site and based on this separation a new ranked gene list was created (see Materials and Methods). (A) Ranking based on promoter proximal peaks within 500bp of TSS and (B) based on the promoter distal peaks outside this 500bp. (C) Ranking based on promoter proximal peaks within 1kb of TSS and (D) based on the promoter distal peaks outside this 1kb. (E) The peak to gene assignment is now purely based on their nearest TSS, this way all peaks are assigned to a gene and for each gene the most significant differential peak is retained to create the ranked gene list. (TIF) [file pgen.1004994.s006.tif]

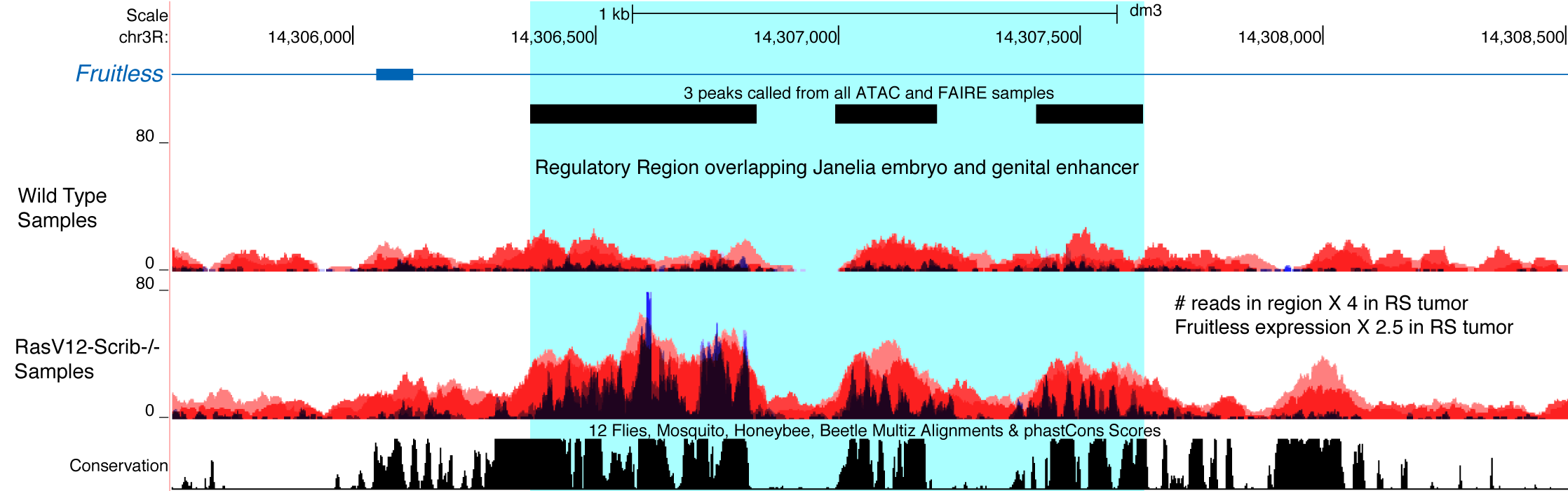

Supplement: S7 Fig — Regulatory region inside an intron of fruitless overlapping with a previously described Janelia genital enhancer (R22G01). The enhancer gains activity in the RS-tumor (4 times more reads) and may underlie the increased transcription of the fruitless gene. (TIF) [file pgen.1004994.s007.tif]
