# Supplementary material for: Discovery of Transcription Factors and Regulatory Regions Driving In Vivo Tumor Development by ATAC-seq and FAIRE-seq Open Chromatin Profiling
Source: PLoS Genet. 2015 Feb 13;11(2):e1004994. doi: 10.1371/journal.pgen.1004994 (PMC4334524; doi:10.1371/journal.pgen.1004994)
Supplement: S1 Table — (DOCX) [file pgen.1004994.s008.docx]

Supplementary Table 1

| **Genes** | **LogFC-Gene** | **Regulatory Region** | **LogFC-Region** | **FisheradjPval** |
| --- | --- | --- | --- | --- |
| CG13215 | 5.34 | chr2R:7127501-7128112 | 2.87 | 1.00E-15 |
| Ilp8 | 4.65 | chr3L:17026996-17027913 | 2.94 | 1.00E-15 |
| PGRP-SA | 4.59 | chrX:11455617-11456016 | 2.02 | 1.00E-15 |
| CG13606 | 4.04 | chr3R:19997246-19997951 | 2.30 | 1.00E-15 |
| yellow-b | 3.26 | chr2L:16754743-16755463 | 2.00 | 1.00E-15 |
| Tdc1 | 3.17 | chr2R:2569358-2569941 | 2.94 | 1.00E-15 |
| CG9572 | 3.03 | chrX:20041968-20042378 | 2.85 | 1.00E-15 |
| Mmp1 | 3.00 | chr2R:20575325-20575586 | 2.07 | 1.00E-15 |
| CG1572 | 2.76 | chrX:11451621-11452041 | 2.28 | 1.00E-15 |
| CG14968 | 2.75 | chr3L:3300125-3301409 | 1.82 | 1.00E-15 |
| Rcd2 | 2.38 | chr3L:20528325-20528846 | 2.67 | 1.00E-15 |
| obst-E | 2.29 | chr2L:5765986-5766401 | 2.13 | 1.00E-15 |
| Ets21C | 2.28 | chr2L:548672-548905 | 3.41 | 1.00E-15 |
| CG10527 | 2.13 | chr2R:16960672-16960869 | 2.75 | 1.00E-15 |
| BBS8 | 1.91 | chr2L:422757-423265 | 2.20 | 1.00E-15 |
| wun | 1.77 | chr2R:5293901-5294283 | 2.84 | 1.00E-15 |
| mfas | 1.56 | chr3R:7815834-7816235 | 2.63 | 1.00E-15 |
| fru | 1.33 | chr3R:14306366-14306832 | 2.58 | 1.00E-15 |
| Snap25 | 1.17 | chr3L:24168669-24168781 | 4.17 | 1.00E-15 |
| l(1)G0232 | 1.12 | chrX:9540782-9541261 | 2.79 | 1.00E-15 |
| Imp | 1.08 | chrX:10702657-10703155 | 2.70 | 1.00E-15 |
| Chd64 | 1.07 | chr3L:4119065-4119492 | 2.92 | 1.00E-15 |
| beg | 0.97 | chr3L:17026996-17027913 | 2.94 | 1.00E-15 |
| Cyp18a1 | 0.74 | chrX:18589829-18590376 | 2.05 | 1.00E-15 |
| Clic | 0.72 | chrX:13709138-13709644 | 2.84 | 1.00E-15 |
| S6k | 0.67 | chr3L:5800815-5801289 | 2.58 | 1.00E-15 |
| aos | 0.67 | chr3L:16471873-16472154 | 3.09 | 1.00E-15 |
| tup | 0.67 | chr2L:18877104-18877593 | 3.03 | 1.00E-15 |
| dia | 0.60 | chr2L:20759330-20759794 | 2.74 | 1.00E-15 |
| Mkp3 | 0.57 | chr3L:19065302-19065971 | 2.70 | 1.00E-15 |
| Sam-S | 0.45 | chr2L:110027-110520 | 2.68 | 1.00E-15 |
| CG9422 | 0.42 | chr2R:2569358-2569941 | 2.94 | 1.00E-15 |
| Bsg | 0.42 | chr2L:8085537-8086150 | 2.92 | 1.00E-15 |
| Tig | 3.33 | chr2L:6423051-6423845 | 2.23 | 6.33E-15 |
| CG1648 | 2.06 | chr2R:5701441-5702329 | 1.39 | 6.33E-15 |
| p53 | 0.73 | chr3R:18876970-18877300 | 3.04 | 6.33E-15 |
| apt | 1.18 | chr2R:19475892-19476032 | 2.74 | 1.72E-14 |
| Fic | 0.97 | chr2L:6423051-6423845 | 2.23 | 1.72E-14 |
| sty | 0.88 | chr3L:3422683-3423159 | 1.36 | 2.79E-14 |
| CG17124 | 2.26 | chr2L:10746149-10746386 | 2.40 | 3.21E-14 |
| CG3746 | 1.46 | chr2R:18537237-18537480 | 2.48 | 3.21E-14 |
| CAP | 0.47 | chr2R:6162952-6163208 | 2.62 | 3.21E-14 |
| CG31955 | 2.90 | chr2L:3712438-3713467 | 1.36 | 4.61E-14 |
| CG10089 | 1.38 | chr3L:13469507-13469995 | 1.95 | 4.61E-14 |
| Tsp42El | 1.88 | chr2R:2933096-2934033 | 1.69 | 5.52E-14 |
| spz3 | 1.22 | chr2L:7875566-7875828 | 2.18 | 5.52E-14 |
| nec | 1.64 | chr2R:3045684-3046218 | 1.69 | 6.77E-14 |
| Idgf4 | 1.18 | chrX:9825941-9826902 | 1.88 | 6.77E-14 |
| insc | 0.93 | chr2R:16727135-16727607 | 2.33 | 9.05E-14 |
| Sulf1 | 1.28 | chr3R:12145754-12146527 | 1.66 | 1.04E-13 |
| Tre1 | 0.77 | chrX:5569114-5569483 | 2.40 | 1.04E-13 |
| upd3 | 1.13 | chrX:18171108-18171510 | 2.02 | 1.11E-13 |
| LamC | 1.02 | chr2R:10462544-10463055 | 1.44 | 1.50E-13 |
| cindr | 0.45 | chr3R:26649343-26649865 | 2.52 | 1.56E-13 |
| CG6753 | 2.67 | chr3R:8471748-8472163 | 1.88 | 2.80E-13 |
| CG1124 | 1.08 | chr3R:789108-789666 | 1.63 | 4.13E-13 |
| Ude | 2.80 | chr3R:20624607-20625774 | 1.64 | 4.79E-13 |
| CG15629 | 1.10 | chr2L:4812732-4813181 | 1.88 | 5.56E-13 |
| CG6330 | 2.51 | chr3R:22778863-22779093 | 1.83 | 7.13E-13 |
| CG9747 | 1.39 | chr3R:26018724-26019198 | 1.78 | 7.97E-13 |
| puc | 0.75 | chr3R:3942721-3943386 | 2.29 | 1.07E-12 |
| chinmo | 2.22 | chr2L:1669207-1669524 | 2.08 | 1.51E-12 |
| PGRP-LB | 0.54 | chr3R:7280484-7280886 | 2.33 | 2.10E-12 |
| CG13624 | 1.18 | chr3R:20396180-20396478 | 2.57 | 3.28E-12 |
| dar1 | 0.72 | chr3L:3640237-3640383 | 2.46 | 4.03E-12 |
| nahoda | 0.69 | chr2R:18784838-18785266 | 2.03 | 5.54E-12 |
| Tsp | 1.52 | chr2L:6694179-6694409 | 1.98 | 7.46E-12 |
| rpr | 0.76 | chr3L:18394934-18395810 | 1.57 | 8.82E-12 |
| CG14567 | 1.99 | chr3L:21732223-21732848 | 1.41 | 9.13E-12 |
| CG13722 | 2.07 | chr3L:4489516-4489863 | 2.03 | 9.59E-12 |
| cher | 1.22 | chr3R:12939561-12939803 | 2.49 | 1.49E-11 |
| Lcp65Ag2 | 3.94 | chr3L:6126605-6126898 | 1.81 | 3.56E-11 |
| CG11073 | 1.05 | chr2R:17986840-17987163 | 1.42 | 4.32E-11 |
| CG5535 | 1.38 | chr3L:17850294-17850882 | 2.18 | 5.08E-11 |
| CG14879 | 1.49 | chr3R:12173465-12173712 | 1.49 | 5.66E-11 |
| CG8066 | 2.41 | chr3R:10394250-10394836 | 1.99 | 5.72E-11 |
| CG13024 | 0.71 | chr3L:16806835-16807201 | 1.69 | 6.81E-11 |
| CG6701 | 1.39 | chr2R:9865860-9866225 | 1.88 | 7.62E-11 |
| CG10916 | 1.01 | chr2R:14058746-14060154 | 1.40 | 9.02E-11 |
| AcCoAS | 1.55 | chr3L:21269778-21270366 | 1.26 | 9.52E-11 |
| Tg | 2.01 | chr2L:8012469-8013273 | 1.65 | 1.05E-10 |
| CG11852 | 2.85 | chr3R:21062181-21062422 | 1.40 | 1.06E-10 |
| CG31673 | 2.94 | chr2L:20830301-20830752 | 1.21 | 1.09E-10 |
| SP1173 | 1.39 | chr3L:6692874-6693176 | 2.35 | 1.21E-10 |
| Clect27 | 2.77 | chr2L:4801764-4802194 | 1.85 | 1.21E-10 |
| CG5001 | 0.71 | chr2L:1194746-1195066 | 2.55 | 1.26E-10 |
| pigs | 1.03 | chrX:6515265-6515615 | 2.10 | 1.29E-10 |
| CG8369 | 2.13 | chr3R:4647500-4647859 | 2.39 | 1.29E-10 |
| CG17108 | 3.44 | chr2L:10694001-10694306 | 2.40 | 1.31E-10 |
| Atf3 | 0.85 | chrX:1169632-1170203 | 1.34 | 1.38E-10 |
| CG7059 | 2.18 | chr3R:18215458-18216587 | 1.84 | 1.42E-10 |
| CG42326 | 2.10 | chr2R:4473681-4474086 | 1.97 | 1.53E-10 |
| Pax | 1.65 | chr2L:19421383-19421518 | 2.49 | 1.74E-10 |
| GstE7 | 1.38 | chr2R:14294224-14294498 | 1.99 | 1.77E-10 |
| CG13124 | 0.98 | chr2L:9906932-9907794 | 1.43 | 3.39E-10 |
| CG12607 | 1.18 | chr3L:4446150-4446607 | 2.04 | 3.52E-10 |
| CG14984 | 1.84 | chr3L:3951912-3952589 | 1.24 | 3.62E-10 |
| CG14608 | 1.15 | chr3R:3053989-3054281 | 1.73 | 3.73E-10 |
| spir | 1.39 | chr2L:20320197-20320877 | 2.20 | 4.53E-10 |
| ftz-f1 | 1.27 | chr3L:18784031-18784852 | 2.27 | 5.05E-10 |
| eg | 1.04 | chr3L:21797560-21798478 | 1.72 | 6.82E-10 |
| sda | 1.33 | chr3R:22752665-22753156 | 1.69 | 7.91E-10 |
| CG15611 | 0.86 | chr2R:12997857-12998246 | 2.01 | 8.29E-10 |
| CG14207 | 1.11 | chrX:19500615-19500963 | 1.88 | 8.43E-10 |
| Dic1 | 0.56 | chr3R:9189201-9189409 | 2.50 | 8.74E-10 |
| Trim9 | 0.70 | chr2L:10611115-10611378 | 1.80 | 9.34E-10 |
| CG5541 | 0.66 | chrX:14942791-14943261 | 1.89 | 9.90E-10 |
| CG7267 | 0.71 | chrX:8971122-8971545 | 1.86 | 1.09E-09 |
| sNPF | 2.99 | chr2L:20027170-20027497 | 1.82 | 1.22E-09 |
| GstE6 | 1.58 | chr2R:14294224-14294498 | 1.99 | 1.29E-09 |
| moody | 0.75 | chrX:1943397-1944880 | 1.41 | 1.54E-09 |
| CG15239 | 1.22 | chrX:3781728-3781979 | 1.46 | 1.71E-09 |
| Tm1 | 1.17 | chr3R:11112500-11112694 | 1.79 | 1.93E-09 |
| Cys | 1.70 | chr3R:10394250-10394836 | 1.99 | 2.44E-09 |
| CG15546 | 0.89 | chr3R:26702270-26702513 | 1.74 | 2.53E-09 |
| CG15117 | 0.78 | chr2R:15013265-15013965 | 1.37 | 3.07E-09 |
| CG8547 | 0.94 | chr2R:10154197-10154638 | 1.31 | 3.69E-09 |
| Naam | 0.66 | chr3R:15513936-15514869 | 1.77 | 3.95E-09 |
| CG17549 | 0.89 | chr2L:19389529-19390252 | 1.32 | 3.95E-09 |
| CG6749 | 1.13 | chr3L:9726743-9727161 | 0.99 | 4.73E-09 |
| Pvf2 | 0.58 | chr2L:7076332-7076739 | 2.05 | 5.13E-09 |
| PrBP | 0.99 | chr2L:8685263-8685527 | 1.70 | 6.22E-09 |
| CG10208 | 1.34 | chr3R:19599586-19600141 | 1.51 | 9.73E-09 |
| Aldh | 1.17 | chr2L:9387529-9387721 | 2.20 | 1.17E-08 |
| Cct1 | 0.85 | chr3L:1548861-1549061 | 2.07 | 1.32E-08 |
| CG8303 | 2.19 | chr2R:12488964-12489531 | 1.10 | 1.36E-08 |
| CG9192 | 1.74 | chr3L:1238197-1238827 | 1.73 | 1.68E-08 |
| mol | 1.62 | chr2L:14999505-14999907 | 1.39 | 2.05E-08 |
| os | 2.47 | chrX:18203128-18203958 | 1.15 | 2.39E-08 |
| CG34227 | 3.00 | chr2R:7135456-7135868 | 1.58 | 2.52E-08 |
| CG15745 | 2.35 | chrX:13196808-13197097 | 1.64 | 2.60E-08 |
| CG14696 | 1.09 | chr3R:6710041-6710490 | 1.23 | 2.73E-08 |
| vir-1 | 3.45 | chr2L:12404427-12405070 | 1.61 | 3.30E-08 |
| CG7054 | 1.82 | chr3R:18290268-18290723 | 1.61 | 3.56E-08 |
| CG33170 | 1.47 | chr3L:22858379-22858584 | 1.66 | 4.51E-08 |
| Sp212 | 1.54 | chr3R:10049506-10050037 | 1.38 | 4.52E-08 |
| CG6171 | 0.70 | chr3R:11188546-11188800 | 2.33 | 4.58E-08 |
| Esyt2 | 1.80 | chr3R:20374578-20374818 | 2.03 | 5.51E-08 |
| atilla | 1.90 | chr2L:12177609-12178021 | 1.53 | 5.71E-08 |
| CG6191 | 1.47 | chr2R:9474756-9475781 | 1.35 | 6.15E-08 |
| CG15784 | 1.15 | chrX:5324429-5324740 | 1.44 | 6.26E-08 |
| CG9336 | 0.72 | chr2L:20858244-20859347 | 1.41 | 6.54E-08 |
| Phk-3 | 1.95 | chr2R:20859211-20859746 | 1.03 | 8.52E-08 |
| CG1635 | 0.97 | chr3R:27414031-27414480 | 1.27 | 1.14E-07 |
| Tsp42Ee | 0.83 | chr2R:2903019-2903577 | 1.32 | 1.22E-07 |
| upd2 | 0.99 | chrX:18139291-18139489 | 2.09 | 1.23E-07 |
| Sk1 | 0.95 | chrX:11336178-11336656 | 1.26 | 1.38E-07 |
| viaf | 1.10 | chr3L:11984981-11985472 | 1.61 | 1.49E-07 |
| Cyp6a20 | 2.37 | chr2R:10768900-10769041 | 2.37 | 1.67E-07 |
| Listericin | 1.75 | chr2R:7131143-7131312 | 1.94 | 1.93E-07 |
| Mpc1 | 0.74 | chr3R:14752805-14753030 | 1.46 | 2.16E-07 |
| tx | 1.11 | chr3R:22263732-22263919 | 1.76 | 4.36E-07 |
| dyn-p25 | 0.74 | chr2L:14999505-14999907 | 1.39 | 4.72E-07 |
| CG12643 | 0.78 | chrX:10157902-10158518 | 1.29 | 5.00E-07 |
| CG12746 | 0.80 | chr3R:1391992-1392208 | 1.63 | 5.95E-07 |
| JhI-26 | 0.73 | chr2R:12176574-12176908 | 1.71 | 6.18E-07 |
| CG2975 | 1.17 | chr2L:2840002-2840734 | 1.00 | 6.42E-07 |
| CG13230 | 1.20 | chr2R:7041281-7041645 | 1.68 | 6.48E-07 |
| CG15279 | 2.50 | chr2L:14850235-14850619 | 2.26 | 7.76E-07 |
| Cpr12A | 1.63 | chrX:13432290-13432713 | 1.26 | 9.42E-07 |
| lsn | 0.66 | chr3R:17690059-17690459 | 1.64 | 1.11E-06 |
| Spn47C | 1.86 | chr2R:6829820-6830029 | 1.46 | 1.14E-06 |
| CG15556 | 1.20 | chr3R:26955940-26956335 | 1.41 | 2.14E-06 |
| CG9836 | 0.65 | chr3R:4648950-4649061 | 1.93 | 2.37E-06 |
| CG33054 | 1.33 | chr3L:21194410-21194575 | 1.44 | 2.93E-06 |
| CG10126 | 2.90 | chr3R:8683202-8683662 | 0.88 | 3.06E-06 |
| CG13046 | 0.99 | chr3L:16281905-16282262 | 1.35 | 3.41E-06 |
| CG7294 | 1.38 | chr2L:10689085-10689488 | 1.20 | 3.84E-06 |
| Idgf3 | 2.67 | chr2L:16450992-16451314 | 1.14 | 4.72E-06 |
| Cyt-c-p | 1.15 | chr2L:16720895-16721407 | 0.89 | 4.76E-06 |
| CG33474 | 2.66 | chr2R:6309354-6309687 | 0.52 | 5.77E-06 |
| CG1890 | 1.91 | chr3R:27569038-27569366 | 0.54 | 5.78E-06 |
| CG33110 | 0.59 | chr3R:18398458-18398801 | 1.71 | 6.05E-06 |
| CG4998 | 1.03 | chr3L:16330735-16330902 | 2.14 | 7.24E-06 |
| CAHbeta | 1.16 | chr3R:4806839-4807137 | 0.91 | 7.83E-06 |
| CG17029 | 1.14 | chr3L:15969990-15970182 | 1.46 | 8.56E-06 |
| Cyp28a5 | 1.21 | chr2L:13978106-13978439 | 1.15 | 9.59E-06 |
| Mlc2 | 1.52 | chr3R:26000203-26000937 | 1.10 | 1.03E-05 |
| Tdc2 | 1.19 | chr2R:2577389-2577715 | 1.47 | 1.19E-05 |
| CG11158 | 1.51 | chrX:13576961-13577066 | 1.35 | 1.21E-05 |
| Spn55B | 1.10 | chr2R:14013728-14014023 | 0.92 | 1.42E-05 |
| Rab9 | 1.72 | chr2L:19433264-19433704 | 1.02 | 1.43E-05 |
| Sps2 | 1.17 | chr2L:10342553-10342804 | 0.99 | 1.60E-05 |
| yellow-c | 1.62 | chr2L:15035853-15036079 | 1.23 | 1.76E-05 |
| CG12344 | 0.62 | chr2R:6721672-6721891 | 1.74 | 2.37E-05 |
| CG34325 | 2.00 | chrX:16615265-16615630 | 0.82 | 2.49E-05 |
| EndoGI | 1.04 | chr2L:16257918-16258092 | 1.04 | 2.58E-05 |
| Spn42Dc | 1.64 | chr2R:2768333-2768588 | 1.21 | 2.62E-05 |
| nrv3 | 0.75 | chr2L:21390203-21390377 | 1.99 | 3.01E-05 |
| CG13117 | 2.48 | chr2L:9736450-9736761 | 0.71 | 3.22E-05 |
| CG13631 | 1.00 | chr3R:20592276-20592578 | 1.58 | 3.38E-05 |
| Tektin-C | 3.62 | chr3L:5790427-5790608 | 0.77 | 3.63E-05 |
| Pvf1 | 1.19 | chrX:18728379-18728508 | 1.39 | 4.08E-05 |
| CG17105 | 0.71 | chr2L:10679925-10680476 | 1.43 | 6.61E-05 |
| CG31098 | 1.74 | chr3R:21118438-21119005 | 0.66 | 7.11E-05 |
| CG1299 | 0.75 | chr3L:4145841-4145999 | 1.90 | 9.73E-05 |
| CG31549 | 1.23 | chr3R:1457940-1458169 | 0.88 | 0.000103176 |
| Cpr47Eb | 2.24 | chr2R:7142171-7142281 | 1.23 | 0.000128042 |
| pinta | 1.40 | chr3R:18249927-18250058 | 0.94 | 0.000142832 |
| CG10559 | 3.14 | chr3R:21141548-21142108 | 0.54 | 0.000154121 |
| CG31370 | 2.88 | chr3R:21129646-21130219 | 0.57 | 0.000157171 |
| Pcp | 1.17 | chr2L:7021522-7021672 | 1.40 | 0.000182021 |
| wun2 | 2.61 | chr2R:5302641-5302947 | 0.49 | 0.00019903 |
| CG7296 | 1.09 | chr2L:10686857-10687371 | 0.92 | 0.000234031 |
| CG4267 | 2.35 | chr2L:2243184-2243322 | 1.20 | 0.000262333 |
| CG33469 | 1.25 | chr2R:10493651-10493876 | 0.90 | 0.000293091 |
| spz5 | 1.12 | chr3L:2890671-2891001 | 0.97 | 0.000315816 |
| y | 1.91 | chrX:250221-250570 | 0.86 | 0.000481331 |
| Ugt35a | 1.43 | chr3R:6998640-6998907 | 0.86 | 0.000496457 |
| CG32196 | 1.09 | chr3L:18453761-18454197 | 1.05 | 0.000518366 |
| Arc1 | 2.10 | chr2R:10245621-10245744 | 0.54 | 0.000559357 |
| CG9743 | 0.89 | chr3R:26023456-26023572 | 1.12 | 0.001237871 |
| CG4957 | 1.16 | chr2L:10268692-10268926 | 1.03 | 0.001941861 |
| CG32364 | 1.37 | chr3L:8203787-8203993 | 0.92 | 0.002183478 |
| Sodh-2 | 1.72 | chr3R:6701618-6701824 | 0.60 | 0.002526926 |
