# Supplementary material for: Discovery of Transcription Factors and Regulatory Regions Driving In Vivo Tumor Development by ATAC-seq and FAIRE-seq Open Chromatin Profiling
Source: PLoS Genet. 2015 Feb 13;11(2):e1004994. doi: 10.1371/journal.pgen.1004994 (PMC4334524; doi:10.1371/journal.pgen.1004994)
Supplement: S2 Table — (DOCX) [file pgen.1004994.s009.docx]

Supplementary Table 2

| **Genes** | **LogFC-Gene** | **Regulatory Region** | **LogFC-Region** | **FisheradjPval** |
| --- | --- | --- | --- | --- |
| ImpE2 | -3.01 | chr3L:3513376-3513774 | -3.49 | 1.00E-15 |
| sm | -2.49 | chr2R:15519336-15519922 | -1.82 | 1.00E-15 |
| CG1273 | -2.37 | chr3L:4270921-4271447 | -2.13 | 1.00E-15 |
| Blimp-1 | -2.35 | chr3L:5623668-5624140 | -1.80 | 1.00E-15 |
| CG9416 | -2.35 | chr2R:15259819-15260968 | -1.97 | 1.00E-15 |
| Eip78C | -2.30 | chr3L:21231416-21232235 | -2.60 | 1.00E-15 |
| CG5065 | -1.99 | chr2R:12498817-12499422 | -2.29 | 1.00E-15 |
| CG11899 | -1.91 | chr3R:25025608-25026357 | -1.81 | 1.00E-15 |
| klu | -1.81 | chr3L:10991463-10992243 | -2.19 | 1.00E-15 |
| CG32447 | -1.71 | chr3L:21703606-21703879 | -2.12 | 1.00E-15 |
| Sema-2b | -1.54 | chr2R:12275400-12275720 | -2.44 | 1.00E-15 |
| mub | -1.37 | chr3L:21876183-21876754 | -1.76 | 1.00E-15 |
| hth | -1.34 | chr3R:6343821-6344734 | -2.26 | 1.00E-15 |
| a | -1.30 | chr2R:18043872-18044575 | -2.51 | 1.00E-15 |
| Syp | -1.28 | chr3R:16595635-16596421 | -1.98 | 1.00E-15 |
| fra | -1.20 | chr2R:8420439-8420847 | -2.18 | 1.00E-15 |
| grh | -1.19 | chr2R:13723639-13724473 | -2.65 | 1.00E-15 |
| Np | -1.12 | chr2R:4891719-4892487 | -2.69 | 1.00E-15 |
| Oatp74D | -1.07 | chr3L:17488751-17489173 | -2.05 | 1.00E-15 |
| CG42389 | -1.06 | chr2L:16597685-16597844 | -2.82 | 1.00E-15 |
| CG34104 | -1.05 | chrX:10008391-10008806 | -2.42 | 1.00E-15 |
| trx | -0.98 | chr3R:10101414-10102038 | -2.45 | 1.00E-15 |
| Eip74EF | -0.97 | chr3L:17604723-17605576 | -1.54 | 1.00E-15 |
| tna | -0.96 | chr3L:10844485-10844954 | -2.39 | 1.00E-15 |
| S | -0.91 | chr2L:1070042-1070460 | -2.29 | 1.00E-15 |
| Reck | -0.83 | chr3L:15000924-15001602 | -2.36 | 1.00E-15 |
| klar | -0.82 | chr3L:437556-437975 | -2.76 | 1.00E-15 |
| Dl | -0.81 | chr3R:15132491-15132797 | -2.10 | 1.00E-15 |
| CG7337 | -0.80 | chr2L:1887422-1888312 | -2.52 | 1.00E-15 |
| CG8213 | -0.70 | chr2R:4870383-4870755 | -2.37 | 1.00E-15 |
| CG33993 | -0.64 | chr3L:5941621-5942230 | -2.12 | 1.00E-15 |
| spri | -0.60 | chrX:10478275-10478625 | -2.00 | 1.00E-15 |
| osp | -0.60 | chr2L:14642201-14642609 | -2.13 | 1.00E-15 |
| Eip63E | -0.59 | chr3L:3513376-3513774 | -3.49 | 1.00E-15 |
| jing | -0.58 | chr2R:2434637-2435401 | -2.09 | 1.00E-15 |
| aay | -0.57 | chr3L:9413359-9413788 | -2.10 | 1.00E-15 |
| sano | -0.55 | chr2R:14829630-14829990 | -2.18 | 1.00E-15 |
| pio | -0.53 | chr2R:20478445-20479096 | -2.31 | 1.00E-15 |
| CG3967 | -0.50 | chr3L:9413359-9413788 | -2.10 | 1.00E-15 |
| CG8177 | -0.48 | chr3L:9760994-9761463 | -2.46 | 1.00E-15 |
| Argk | -0.98 | chr3L:9049551-9050046 | -1.81 | 6.33E-15 |
| bab1 | -0.96 | chr3L:1095917-1096406 | -2.03 | 6.33E-15 |
| hbs | -0.63 | chr2R:10921151-10921982 | -1.68 | 6.33E-15 |
| St2 | -1.81 | chr3R:5077325-5077696 | -2.10 | 6.33E-15 |
| ana | -1.45 | chr2R:4951556-4952211 | -1.96 | 1.21E-14 |
| Dys | -0.56 | chr3R:15397738-15398193 | -2.08 | 1.21E-14 |
| CG3831 | -0.93 | chr2R:18829567-18830156 | -2.04 | 2.27E-14 |
| Ser | -0.58 | chr3R:23001704-23002321 | -2.45 | 2.79E-14 |
| CG15628 | -1.64 | chr2L:4820476-4821020 | -1.65 | 3.21E-14 |
| rho | -0.81 | chr3L:1458430-1458825 | -2.09 | 3.21E-14 |
| ed | -0.81 | chr2L:4068889-4069640 | -2.44 | 3.71E-14 |
| CG30497 | -0.79 | chr2R:3640005-3640460 | -2.34 | 4.19E-14 |
| mew | -0.82 | chrX:13140170-13140833 | -2.17 | 6.35E-14 |
| pk | -1.55 | chr2R:3082406-3082957 | -2.31 | 6.35E-14 |
| sba | -0.94 | chr3R:19733827-19734741 | -1.62 | 1.18E-13 |
| pdgy | -1.35 | chrX:14845090-14845342 | -1.85 | 1.26E-13 |
| bib | -1.29 | chr2L:9986662-9986901 | -1.64 | 1.46E-13 |
| CG4928 | -0.92 | chrX:16824283-16824566 | -2.33 | 1.82E-13 |
| pum | -1.14 | chr3R:4960908-4961360 | -2.28 | 2.18E-13 |
| tmod | -0.82 | chr3R:26393224-26393498 | -2.51 | 2.18E-13 |
| LM408 | -1.60 | chr2L:10457389-10457969 | -1.97 | 2.77E-13 |
| Alh | -1.30 | chr3R:2934804-2935111 | -1.65 | 3.64E-13 |
| Os-C | -1.98 | chr3R:3804110-3804841 | -2.23 | 4.13E-13 |
| dyl | -1.10 | chr3L:4304289-4305184 | -2.71 | 5.65E-13 |
| CG9134 | -0.73 | chr3L:1269029-1269406 | -2.05 | 5.70E-13 |
| CG9005 | -1.15 | chr2R:7517092-7517768 | -1.33 | 6.68E-13 |
| CG42342 | -1.11 | chr3R:12348931-12349315 | -2.12 | 8.39E-13 |
| fz | -0.99 | chr3L:14341214-14341480 | -1.83 | 8.51E-13 |
| CG3823 | -2.93 | chrX:6199511-6199977 | -2.20 | 9.61E-13 |
| hdc | -0.64 | chr3R:26110458-26111310 | -1.74 | 1.04E-12 |
| SP1029 | -1.60 | chr3R:25074930-25075144 | -2.24 | 1.16E-12 |
| Spn43Aa | -3.70 | chr2R:3035044-3035235 | -2.26 | 1.49E-12 |
| Pka-C3 | -0.54 | chr3L:15934113-15934654 | -2.20 | 1.56E-12 |
| Fas3 | -0.88 | chr2L:18351418-18351995 | -1.82 | 1.81E-12 |
| dy | -1.08 | chrX:11672722-11673350 | -2.42 | 1.82E-12 |
| Cyp310a1 | -0.96 | chr2L:18654001-18654881 | -1.79 | 2.01E-12 |
| CG34347 | -1.12 | chr3R:27148100-27148656 | -1.85 | 2.15E-12 |
| ltd | -1.36 | chr2R:5069207-5069592 | -1.70 | 2.16E-12 |
| dsx | -2.27 | chr3R:3780034-3780469 | -2.14 | 2.36E-12 |
| gk | -0.82 | chr3L:18130530-18130810 | -2.37 | 2.46E-12 |
| sev | -1.56 | chrX:10972959-10973868 | -1.84 | 2.53E-12 |
| CG6280 | -0.87 | chr2R:9637376-9637973 | -2.15 | 2.64E-12 |
| CG11147 | -1.09 | chr2L:5738774-5739602 | -2.36 | 3.47E-12 |
| gukh | -1.07 | chr3R:14819906-14820531 | -1.65 | 4.06E-12 |
| CD98hc | -0.56 | chr3R:3804110-3804841 | -2.23 | 4.47E-12 |
| CG30463 | -2.15 | chr2R:12620804-12621042 | -2.08 | 4.68E-12 |
| CG3376 | -1.05 | chr2R:20149173-20149495 | -1.86 | 7.16E-12 |
| bab2 | -2.32 | chr3L:1167094-1167528 | -1.71 | 7.68E-12 |
| toe | -0.94 | chr3L:12430098-12430362 | -1.93 | 8.07E-12 |
| RhoGAP19D | -1.14 | chrX:20361559-20362090 | -1.42 | 9.69E-12 |
| Hsp67Ba | -3.38 | chr3L:9372814-9373821 | -1.47 | 1.12E-11 |
| arr | -1.79 | chr2R:9361819-9362171 | -1.65 | 1.36E-11 |
| CG7080 | -1.00 | chr3R:18149749-18150595 | -1.85 | 1.61E-11 |
| CG42240 | -2.08 | chrX:6225808-6226253 | -1.65 | 1.90E-11 |
| nyo | -1.77 | chr3R:27368401-27369217 | -1.89 | 2.39E-11 |
| Cralbp | -0.97 | chr3L:5779554-5780277 | -1.68 | 2.60E-11 |
| Hr39 | -0.72 | chr2L:21245087-21245647 | -1.43 | 2.60E-11 |
| Cpr97Ea | -2.60 | chr3R:22910315-22910513 | -2.07 | 2.77E-11 |
| CG15630 | -0.79 | chr2L:4791800-4792375 | -1.47 | 3.43E-11 |
| CG15905 | -2.91 | chr2R:15534454-15535501 | -1.73 | 3.53E-11 |
| CBP | -1.21 | chrX:7230840-7231058 | -1.58 | 3.66E-11 |
| CG5758 | -1.76 | chr2L:18121305-18122139 | -1.88 | 4.49E-11 |
| eya | -0.80 | chr2L:6537797-6538153 | -1.73 | 4.72E-11 |
| mim | -0.90 | chr2R:2827463-2827917 | -1.55 | 5.00E-11 |
| CG13284 | -0.78 | chr2L:16844558-16844924 | -1.86 | 5.08E-11 |
| CG32645 | -1.22 | chrX:13036032-13036345 | -2.09 | 5.56E-11 |
| Oatp30B | -1.93 | chr2L:9527402-9528264 | -1.28 | 7.77E-11 |
| Sb | -2.19 | chr3R:11961410-11961991 | -2.07 | 8.84E-11 |
| mld | -0.49 | chr3R:20421140-20421660 | -2.06 | 9.62E-11 |
| CG7720 | -1.94 | chr3R:14613796-14614901 | -1.73 | 1.05E-10 |
| Nep2 | -2.03 | chr3R:555560-555841 | -1.63 | 1.15E-10 |
| Hsp23 | -1.77 | chr3L:9372814-9373821 | -1.47 | 1.22E-10 |
| gem | -0.88 | chr2R:6019877-6020437 | -1.79 | 1.39E-10 |
| bon | -0.81 | chr3R:16420394-16420715 | -1.64 | 1.66E-10 |
| CG32639 | -0.54 | chrX:13132241-13132565 | -2.09 | 1.78E-10 |
| rols | -1.16 | chr3L:12033072-12034009 | -1.52 | 1.89E-10 |
| CG13646 | -1.31 | chr3R:20773101-20773334 | -1.84 | 1.89E-10 |
| CG5873 | -1.45 | chr3R:13258800-13259213 | -1.99 | 2.06E-10 |
| CG2556 | -0.92 | chrX:12401876-12402207 | -1.63 | 2.17E-10 |
| CG13064 | -1.46 | chr3L:16273068-16273647 | -1.34 | 2.28E-10 |
| Nplp1 | -2.45 | chr2R:20918461-20918878 | -1.38 | 2.40E-10 |
| CG6287 | -2.02 | chr2L:11154880-11155225 | -1.44 | 2.47E-10 |
| CG17754 | -0.95 | chrX:9146025-9146523 | -1.37 | 2.90E-10 |
| px | -0.99 | chr2R:18422144-18422604 | -2.04 | 3.29E-10 |
| E23 | -1.41 | chr2L:3341230-3341469 | -2.04 | 3.35E-10 |
| B4 | -0.73 | chr2L:13536315-13536980 | -1.50 | 3.62E-10 |
| nmo | -1.31 | chr3L:7987296-7987552 | -2.20 | 4.18E-10 |
| TwdlT | -1.73 | chr3R:22874363-22874975 | -2.01 | 4.46E-10 |
| CG31475 | -1.12 | chr3R:15008672-15008996 | -1.71 | 4.48E-10 |
| CG11382 | -1.04 | chrX:1102323-1102862 | -1.65 | 4.66E-10 |
| CG10211 | -1.36 | chr2L:18522394-18522929 | -1.35 | 5.26E-10 |
| retn | -1.51 | chr2R:19523239-19523569 | -1.49 | 6.63E-10 |
| CG10657 | -1.55 | chr3L:12487892-12488572 | -1.58 | 7.70E-10 |
| Wnt2 | -1.58 | chr2R:5383956-5384129 | -2.14 | 8.07E-10 |
| Spn100A | -2.33 | chr3R:26515610-26516532 | -1.73 | 8.55E-10 |
| CG7510 | -0.86 | chr3L:17618871-17619266 | -1.47 | 8.94E-10 |
| CG9238 | -2.03 | chr3L:14537597-14537873 | -2.03 | 1.01E-09 |
| MYPT-75D | -1.37 | chr3L:18620590-18621228 | -1.12 | 1.03E-09 |
| mey | -0.77 | chr3R:27324685-27325164 | -1.74 | 1.63E-09 |
| CG3842 | -1.49 | chrX:6237302-6237480 | -1.97 | 1.78E-09 |
| CG14946 | -1.00 | chr2L:12065936-12066247 | -1.96 | 1.82E-09 |
| ImpE3 | -2.97 | chr3R:3855097-3855424 | -1.50 | 1.82E-09 |
| Osi4 | -1.62 | chr3R:2051618-2051976 | -1.87 | 1.85E-09 |
| CG11905 | -1.86 | chr3L:16929605-16929929 | -1.33 | 1.93E-09 |
| CG8170 | -1.30 | chr2R:4915701-4916792 | -1.10 | 2.22E-09 |
| obst-A | -1.37 | chrX:20109766-20110193 | -1.40 | 2.87E-09 |
| Cpr51A | -1.34 | chr2R:10407535-10407945 | -1.39 | 3.02E-09 |
| ect | -0.61 | chr3L:10194596-10195183 | -1.98 | 3.11E-09 |
| pico | -0.64 | chrX:19737151-19737463 | -1.86 | 3.63E-09 |
| otk | -0.75 | chr2R:7900508-7900861 | -1.84 | 3.90E-09 |
| ara | -0.59 | chr3L:12583552-12584265 | -1.99 | 4.55E-09 |
| Cpr65Ec | -0.60 | chr3L:7084201-7085063 | -1.79 | 4.62E-09 |
| CG32676 | -1.01 | chrX:10636252-10636421 | -1.54 | 4.90E-09 |
| CG9990 | -1.27 | chr3R:24505312-24505610 | -1.34 | 5.59E-09 |
| CG5639 | -1.53 | chr3R:23418417-23419729 | -1.61 | 5.67E-09 |
| wgn | -0.68 | chrX:18527501-18527812 | -1.56 | 6.24E-09 |
| CG3097 | -1.14 | chrX:5664760-5665182 | -1.44 | 6.49E-09 |
| tutl | -0.75 | chr2L:4287340-4287661 | -1.47 | 7.50E-09 |
| CG31559 | -1.47 | chr3R:1982486-1983329 | -1.27 | 1.00E-08 |
| CG2747 | -0.83 | chr3R:3855097-3855424 | -1.50 | 1.19E-08 |
| pip | -2.38 | chr3L:19327836-19328102 | -1.69 | 1.31E-08 |
| ari-2 | -0.93 | chr2R:18101580-18102023 | -1.18 | 1.60E-08 |
| CG2150 | -0.58 | chr3R:27593654-27594025 | -1.87 | 1.62E-08 |
| DAAM | -1.05 | chrX:1208683-1209211 | -1.98 | 1.66E-08 |
| Kdm4B | -0.59 | chr2R:9083163-9083867 | -1.79 | 2.20E-08 |
| CG34380 | -0.57 | chr2L:6192665-6193080 | -1.84 | 2.47E-08 |
| Zir | -0.75 | chr2L:92040-92488 | -1.52 | 2.68E-08 |
| GV1 | -0.71 | chr3L:1822235-1822798 | -1.58 | 3.80E-08 |
| CG8420 | -1.98 | chr3R:5072428-5072741 | -1.08 | 3.88E-08 |
| so | -0.84 | chr2R:3312335-3312928 | -1.67 | 3.90E-08 |
| Sobp | -0.82 | chr2R:7718499-7719032 | -1.68 | 4.29E-08 |
| CG6026 | -0.83 | chr3R:14862081-14862791 | -1.49 | 4.31E-08 |
| beat-IIIc | -1.02 | chr2L:17236952-17237880 | -1.41 | 4.52E-08 |
| drl | -0.96 | chr2L:19201380-19202022 | -1.66 | 4.52E-08 |
| sls | -0.75 | chr3L:2046843-2047999 | -1.48 | 5.41E-08 |
| alpha-Man-IIb | -0.79 | chr3R:11632525-11633291 | -1.42 | 6.42E-08 |
| CG15822 | -1.50 | chr3L:2161991-2162142 | -1.71 | 7.10E-08 |
| CG11873 | -1.04 | chr3R:24896250-24896531 | -1.52 | 7.31E-08 |
| CG13737 | -0.89 | chr3L:13871880-13872214 | -1.79 | 8.56E-08 |
| CG34382 | -0.89 | chr3L:9875023-9875458 | -1.51 | 9.73E-08 |
| CG10960 | -2.56 | chr3L:12825295-12826100 | -0.83 | 1.17E-07 |
| CadN | -0.99 | chr2L:17743569-17744164 | -1.30 | 1.38E-07 |
| CG16885 | -1.91 | chr2L:13935951-13936395 | -1.49 | 2.13E-07 |
| Sox102F | -1.29 | chr4:849046-849318 | -1.74 | 2.15E-07 |
| H15 | -0.96 | chr2L:5414621-5415200 | -1.26 | 2.28E-07 |
| Mrtf | -0.93 | chr3L:2735017-2735600 | -1.27 | 2.29E-07 |
| tnc | -1.10 | chr3R:20836064-20836612 | -1.69 | 2.34E-07 |
| Hers | -1.04 | chrX:19771084-19771632 | -1.04 | 2.58E-07 |
| CG1815 | -1.07 | chr3R:27498286-27498785 | -1.19 | 2.65E-07 |
| CG7906 | -1.66 | chr3L:14229647-14229801 | -1.54 | 2.75E-07 |
| Ptp99A | -1.49 | chr3R:25286199-25286628 | -1.15 | 3.06E-07 |
| CG9914 | -0.78 | chrX:16202190-16202705 | -1.42 | 3.94E-07 |
| CG32091 | -1.27 | chr3L:11624382-11624644 | -1.42 | 3.98E-07 |
| CG15282 | -2.86 | chr2L:14711626-14711984 | -1.21 | 4.26E-07 |
| dac | -0.73 | chr2L:16474698-16475083 | -1.41 | 4.28E-07 |
| CG18549 | -0.97 | chr3R:8280119-8280654 | -1.11 | 4.30E-07 |
| CrebA | -1.37 | chr3L:15537801-15538433 | -0.92 | 4.44E-07 |
| CG11353 | -1.08 | chr3L:4532754-4533550 | -1.24 | 4.61E-07 |
| mnb | -1.00 | chrX:17767459-17767748 | -1.57 | 4.66E-07 |
| CG1632 | -1.15 | chrX:8156028-8156271 | -1.60 | 4.68E-07 |
| GNBP3 | -1.28 | chr3L:8947958-8948312 | -1.11 | 5.46E-07 |
| shf | -1.32 | chrX:6669401-6669815 | -1.24 | 5.55E-07 |
| for | -1.21 | chr2L:3632807-3633234 | -1.06 | 5.61E-07 |
| Dll | -1.30 | chr2R:20708337-20708580 | -1.37 | 5.76E-07 |
| CG9935 | -0.91 | chr4:659619-659881 | -1.41 | 5.79E-07 |
| wit | -1.38 | chr3L:4070234-4071114 | -0.91 | 5.79E-07 |
| Osi6 | -2.06 | chr3R:2055137-2055639 | -1.67 | 6.07E-07 |
| l(3)neo38 | -0.79 | chr3R:7601147-7601258 | -1.68 | 7.09E-07 |
| neur | -0.69 | chr3R:4851939-4852188 | -1.56 | 7.23E-07 |
| Cda4 | -1.03 | chrX:22032042-22032547 | -1.62 | 7.46E-07 |
| Lim3 | -1.24 | chr2L:19090756-19091381 | -1.09 | 7.70E-07 |
| CG4408 | -1.74 | chr3R:19193736-19194100 | -1.44 | 7.96E-07 |
| ac | -0.86 | chrX:260661-261005 | -1.45 | 7.98E-07 |
| CG10011 | -0.88 | chr3R:24449575-24450078 | -1.48 | 8.40E-07 |
| robo3 | -0.89 | chr2L:1264824-1265505 | -1.14 | 9.03E-07 |
| CG14476 | -0.79 | chrX:21476554-21477057 | -1.32 | 9.06E-07 |
| Obp99a | -1.46 | chr3R:25496587-25496769 | -1.38 | 1.07E-06 |
| rst | -0.79 | chrX:2855272-2855510 | -1.73 | 1.31E-06 |
| Hsp27 | -1.96 | chr3L:9376063-9376951 | -0.90 | 1.41E-06 |
| lola | -1.35 | chr2R:6405569-6405779 | -1.33 | 1.57E-06 |
| kon | -0.96 | chr2L:18500349-18500675 | -1.25 | 1.64E-06 |
| CG4914 | -2.83 | chr3L:14670409-14670923 | -0.88 | 1.65E-06 |
| zfh2 | -0.88 | chr4:530491-530722 | -1.19 | 1.79E-06 |
| CG13699 | -0.84 | chr3L:18155356-18155808 | -1.34 | 2.25E-06 |
| bip1 | -1.81 | chr3L:8048940-8049141 | -0.95 | 2.46E-06 |
| CG14372 | -1.01 | chr3R:9278106-9278600 | -1.28 | 2.51E-06 |
| Dr | -0.88 | chr3R:25389033-25389365 | -1.70 | 2.73E-06 |
| bowl | -1.12 | chr2L:3770186-3770469 | -1.25 | 3.00E-06 |
| Nep1 | -0.81 | chrX:5989380-5989859 | -1.40 | 3.10E-06 |
| CG13822 | -1.10 | chr3R:19498990-19499160 | -1.61 | 3.12E-06 |
| emp | -0.96 | chr2R:20872400-20872627 | -1.37 | 3.24E-06 |
| Sox21b | -1.02 | chr3L:14121761-14121927 | -1.48 | 3.39E-06 |
| gl | -0.93 | chr3R:14201944-14202147 | -1.55 | 3.70E-06 |
| futsch | -0.80 | chrX:1305093-1306248 | -1.67 | 3.82E-06 |
| CG4586 | -1.86 | chrX:6863320-6864193 | -0.99 | 3.98E-06 |
| CG9312 | -1.04 | chr3R:9591098-9591692 | -1.18 | 4.49E-06 |
| PGRP-LC | -1.37 | chr3L:9332802-9333106 | -1.41 | 5.14E-06 |
| CG7896 | -1.62 | chr3R:25809606-25809970 | -1.06 | 5.25E-06 |
| Ppn | -1.02 | chr3R:24357339-24358254 | -1.35 | 5.67E-06 |
| CG9896 | -2.05 | chr2R:18885953-18886674 | -0.95 | 5.88E-06 |
| stan | -1.04 | chr2R:6591344-6591641 | -1.37 | 6.53E-06 |
| CG30080 | -1.49 | chr2R:11590776-11591001 | -1.10 | 8.26E-06 |
| ImpE1 | -3.00 | chr3L:8365034-8365605 | -0.99 | 8.34E-06 |
| CG3961 | -1.32 | chr3L:18883473-18883827 | -0.87 | 9.92E-06 |
| CG9503 | -1.69 | chrX:14816891-14817968 | -0.80 | 1.03E-05 |
| CG17646 | -1.00 | chr2L:1744902-1745194 | -1.29 | 1.12E-05 |
| Cad88C | -0.71 | chr3R:10459012-10459270 | -1.50 | 1.22E-05 |
| Sdc | -0.70 | chr2R:17359793-17360153 | -1.49 | 1.28E-05 |
| olf186-F | -0.69 | chr2R:13732873-13733167 | -1.55 | 1.39E-05 |
| fog | -0.91 | chrX:22231814-22232195 | -1.42 | 1.80E-05 |
| CG11594 | -2.04 | chr3L:4025854-4026080 | -1.02 | 1.91E-05 |
| Gr64a | -2.02 | chr3L:4025854-4026080 | -1.02 | 2.01E-05 |
| fbp | -1.77 | chr2L:19763894-19764303 | -0.80 | 2.09E-05 |
| spz6 | -1.60 | chr2R:20644577-20645589 | -0.94 | 2.33E-05 |
| sens | -1.60 | chr3L:13391553-13391946 | -1.03 | 2.43E-05 |
| Actn | -0.67 | chrX:1925116-1925639 | -1.50 | 2.53E-05 |
| gogo | -1.11 | chr3L:20286118-20286466 | -1.00 | 2.71E-05 |
| CG30101 | -0.92 | chr2R:13297634-13297971 | -1.20 | 2.78E-05 |
| CG14332 | -1.00 | chr3R:13249914-13250336 | -1.41 | 2.85E-05 |
| CdGAPr | -1.25 | chr2L:19790173-19790537 | -0.88 | 2.86E-05 |
| Mbs | -0.60 | chr3L:16048658-16048772 | -1.74 | 3.58E-05 |
| Obp99b | -2.01 | chr3R:25544325-25544652 | -1.52 | 3.83E-05 |
| CG13082 | -1.23 | chr2L:19538320-19538516 | -1.30 | 5.33E-05 |
| CG17018 | -0.66 | chr2L:22336131-22336314 | -2.09 | 5.43E-05 |
| CG34401 | -1.00 | chrX:18760435-18760775 | -1.07 | 6.26E-05 |
| CG10082 | -1.04 | chr2R:17537351-17537549 | -1.06 | 6.82E-05 |
| DAT | -0.92 | chr2R:12451607-12451859 | -1.28 | 7.01E-05 |
| bnb | -1.70 | chrX:18692195-18692807 | -0.65 | 7.04E-05 |
| neo | -1.65 | chr3R:25644366-25645418 | -0.67 | 9.17E-05 |
| CG12481 | -1.59 | chrX:14080926-14081513 | -1.13 | 9.59E-05 |
| 18w | -1.08 | chr2R:15998065-15998218 | -1.11 | 0.000108291 |
| Cht3 | -1.99 | chr2L:22921777-22921933 | -1.15 | 0.000128179 |
| capu | -1.00 | chr2L:3882494-3882780 | -1.01 | 0.000142123 |
| Osi24 | -1.77 | chr3R:2026998-2027335 | -1.02 | 0.000146354 |
| CG4822 | -1.49 | chr2L:119345-119561 | -1.06 | 0.000198648 |
| yellow-e2 | -1.58 | chr3R:9232678-9232902 | -0.94 | 0.000287919 |
| Cht5 | -1.92 | chr3R:9592808-9593339 | -0.74 | 0.000450533 |
| CG3649 | -2.04 | chr2R:18733271-18734084 | -0.80 | 0.000625548 |
| CG11370 | -2.28 | chr3L:22701647-22701958 | -0.80 | 0.000978927 |
| eyg | -1.39 | chr3L:12461600-12461719 | -0.78 | 0.001602317 |
| run | -1.89 | chrX:20565139-20565483 | -0.66 | 0.001860248 |
| sad | -1.71 | chr3R:7698668-7698784 | -0.79 | 0.006496427 |
