# Supplementary material for: Discovery of Transcription Factors and Regulatory Regions Driving In Vivo Tumor Development by ATAC-seq and FAIRE-seq Open Chromatin Profiling
Source: PLoS Genet. 2015 Feb 13;11(2):e1004994. doi: 10.1371/journal.pgen.1004994 (PMC4334524; doi:10.1371/journal.pgen.1004994)
Supplement: S3 Table — (DOCX) [file pgen.1004994.s010.docx]

Supplementary Table 3

| **Enhancer sets enriched for regions opening in tumor** | **NES** | **NOM p-val** | **FDR q-val** | **FWER p-val** |
| --- | --- | --- | --- | --- |
| VDRC_LEADING_EDGE_CELL | 2.13 | 0.000 | 0.000 | 0.000 |
| VDRC_DORSAL_EPIDERMIS_PRIMORDIUM_BROAD | 2.13 | 0.000 | 0.000 | 0.000 |
| VDRC_ANTERIOR_MIDGUT_PRIMORDIUM | 1.98 | 0.000 | 0.002 | 0.004 |
| REDFLY_TESTIS | 1.98 | 0.000 | 0.002 | 0.004 |
| VDRC_DORSAL_EPIDERMIS_ANLAGE_BROAD | 1.95 | 0.000 | 0.002 | 0.008 |
| VDRC_HEAD_EPIDERMIS_LATERAL_ANLAGE | 1.95 | 0.000 | 0.002 | 0.008 |
| VDRC_PAIR_RULE | 1.91 | 0.004 | 0.003 | 0.016 |
| VDRC_VENTRAL_EPIDERMIS_ANLAGE_BROAD | 1.86 | 0.004 | 0.006 | 0.034 |
| REDFLY_ENDODERM | 1.85 | 0.000 | 0.005 | 0.034 |
| VDRC_POSTERIOR_MIDGUT_ANLAGE | 1.83 | 0.000 | 0.006 | 0.042 |
| VDRC_POSTERIOR_MIDGUT_PRIMORDIUM | 1.83 | 0.000 | 0.006 | 0.044 |
| REDFLY_DORSAL_ECTODERM | 1.78 | 0.000 | 0.010 | 0.076 |
| VDRC_MACROPHAGE | 1.75 | 0.000 | 0.013 | 0.116 |
| JANELIA_GENITAL | 1.73 | 0.000 | 0.017 | 0.152 |
| **Enhancer sets enriched for regions closing in tumor** | **NES** | **NOM p-val** | **FDR q-val** | **FWER p-val** |
| VDRC_ANTERIOR_VENTRAL_NERVE_CORD_SUBSET | -1.67 | 0 | 0.111 | 0.232 |
| VDRC_ANTERIOR_VENTRAL_NERVE_CORD_PRIMORDIUM_SUBSET | -1.57 | 0.009 | 0.136 | 0.494 |
